# Golgi membrane fission requires the CtBP1-S/BARS-induced activation of lysophosphatidic acid acyltransferase δ

**DOI:** 10.1038/ncomms12148

**Published:** 2016-07-12

**Authors:** Alessandro Pagliuso, Carmen Valente, Lucia Laura Giordano, Angela Filograna, Guiling Li, Diego Circolo, Gabriele Turacchio, Vincenzo Manuel Marzullo, Luigi Mandrich, Mikhail A. Zhukovsky, Fabio Formiggini, Roman S. Polishchuk, Daniela Corda, Alberto Luini

**Affiliations:** 1 Institute of Protein Biochemistry, National Research Council, Via Pietro Castellino 111, 80131 Naples, Italy; 2 Telethon Institute of Genetics and Medicine (TIGEM), Via Campi Flegrei 34, Pozzuoli 80078, Italy; 3 IRCCS SDN Istituto di Ricerca Diagnostica e Nucleare, Via Emanuele Gianturco 113, 80143 Naples, Italy; 4 Italian Institute of Technology, Centre for Advanced Biomaterials for Health Care at CRIB, Largo Barsanti e Matteucci 53, Naples 80125, Italy

## Abstract

Membrane fission is an essential cellular process by which continuous membranes split into separate parts. We have previously identified CtBP1-S/BARS (BARS) as a key component of a protein complex that is required for fission of several endomembranes, including basolateral post-Golgi transport carriers. Assembly of this complex occurs at the Golgi apparatus, where BARS binds to the phosphoinositide kinase PI4KIIIβ through a 14-3-3γ dimer, as well as to ARF and the PKD and PAK kinases. We now report that, when incorporated into this complex, BARS binds to and activates a trans-Golgi lysophosphatidic acid (LPA) acyltransferase type δ (LPAATδ) that converts LPA into phosphatidic acid (PA); and that this reaction is essential for fission of the carriers. LPA and PA have unique biophysical properties, and their interconversion might facilitate the fission process either directly or indirectly (via recruitment of proteins that bind to PA, including BARS itself).

## Abstract (web summary)

CtBP1-S/BARS is required for fission of endomembrane compartments including the Golgi. Here the authors show that CtBP1-S/BARS activates a *trans*-Golgi lysophosphatidic acid acyltransferase that catalyses the production of phosphatidic acid and is required for fission of the post-Golgi carrier membrane.

## Graphical abstract

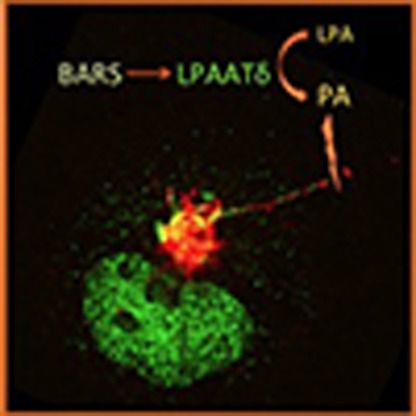

Membrane fission consists of a series of molecular rearrangements by which a tubular or neck-like bilayer joining two membranous compartments undergoes constriction and splits in two parts without leakage of contents. Fission is required for fundamental cellular processes such as the formation of transport vesicles during membrane traffic, organelle partitioning, cell division and in general for the maintenance of the compartmental organization of endomembranes. The mechanisms of fission have been studied intensely during the last decade, and multiple pathways leading to fission have been documented or proposed^1^,^2^,^3^,^4^,^5^. The best characterized fission processes are based on constriction and destabilization of membranes by the mechano-enzyme dynamin^6^,^7^,^8^,^9^,^10^, shallow membrane insertion of amphipathic protein domains^2^,^11^,^12^ and phase separation of lipid domains^3^,^13^. Nevertheless, key aspects of the lipid rearrangements leading to membrane fission remain elusive, and further analysis is required.

We have identified the protein CtBP1-S/BARS (henceforth, BARS) as a key player in the fission of post-Golgi tubular/pleiomorphic carriers^5^,^14^,^15^,^16^, macropinosomes^17^,^18^, COPI-dependent transport vesicles^19^,^20^,^21^ and in the Golgi ribbon partitioning during mitosis^22^,^23^. BARS (brefeldin A ADP-ribosylation substrate) is a member of the C-terminal-binding protein (CtBP) family, which evolutionarily derives from an ancestral dehydrogenase by gene duplication and functional differentiation into proteins involved in transcription, membrane transport, microtubule organization and synaptic transmission^16^. BARS itself is a dual-function protein that controls fission in the cytoplasm and gene transcription in the nucleus^16^,^24^. Structurally, BARS closely resembles the D-hydroxyacid dehydrogenases^25^ and features a ‘classical' NAD(H)-binding Rossman fold^26^, which regulates the interconversion of BARS between a monomeric and a dimeric conformation depending on binding to NAD(H) and/or other ligands to the Rossman domain^16^,^17^,^26^,^27^,^28^. This conversion is critical for function because BARS can drive fission as a monomer, while it is fission-incompetent as a dimer^17^,^19^,^26^,^28^.

The mechanism of action of BARS in fission has been studied mostly in the context of the process of basolateral post-Golgi carrier formation^14^,^15^,^16^. Here BARS assembles into a complex that includes ARF, frequenin (also known as NCS-1), the phosphoinositide kinase PI4KIIIβ, 14-3-3γ and the kinases PKD and PAK, and functions to couple the budding of carriers with fission^15^,^16^. To induce fission, BARS must bind to 14-3-3γ through a phosphorylated serine (Ser147) in its dimerization surface^15^,^16^ (see also below). This binding locks BARS in its monomeric fission-competent conformation. However, how the 14-3-3γ-bound BARS leads to the lipid rearrangements leading to fission remains unclear.

We have previously proposed that BARS-dependent fission involves a lysophosphatidic acid (LPA) acyltransferase (LPAAT) activity, based on the following observations: an LPAAT activity is present in liver Golgi membranes which, on addition of suitable substrates, generates phosphatidic acid (PA)^5^; PA production induces, or correlates with, the fission of Golgi membranes^5^; the addition of BARS to these Golgi membranes stimulates both PA production and membrane fission^5^; treatments that inhibit the formation of monomeric fission-competent BARS inhibit both BARS-stimulated LPAAT activity and membrane fission^5^,^19^,^28^. We have also shown that recombinant BARS is associated with a slow LPAAT activity^5^ (see below) which is inhibited by anti-BARS antibodies, and proposed that this activity is ascribable to BARS itself^5^. This activity, however, was later shown not to be intrinsic to BARS^29^. The simplest interpretation of these collective findings is that BARS binds to and stimulates an endogenous LPAAT, and that this reaction is involved in membrane fission.

Here we have examined this hypothesis. There are 11 known lysophospholipid acyltransferases (LPLATs), four of which have been cloned and shown to transfer fatty acids from acyl-CoA to the *sn-2* position of LPA to form PA (LPAATα, β, γ and δ), while others have mixed specificities for LPA and glycerol phosphate^30^,^31^. We find that (i) BARS interacts with LPAAT type δ (LPAATδ, also known as AGPAT4); (ii) this LPAAT localizes to the *trans*-Golgi and to post-Golgi carrier precursors at the *trans*-Golgi Network (TGN); (iii) the catalytic activity of LPAATδ is essential for Golgi carrier fission; (iv) BARS potently stimulates LPAATδ, and this stimulation is essential for carrier fission; (v) BARS needs to be incorporated into the PI4KIIIβ–14-3-3γ dimer–BARS complex^15^ to stimulate LPAATδ and induce fission. BARS thus appears to function as an adaptor/regulator protein that binds to and stimulates LPAATδ, to induce LPA–PA conversion and carrier fission. LPA and PA have unique biophysical properties that can markedly affect the organization of lipid bilayers^32^. Their interconversion might facilitate the fission process either directly or through the action of PA-binding proteins, including BARS itself^21^.

## Results

### LPAATδ localizes at the Golgi and binds directly to BARS

**Figure 1:**
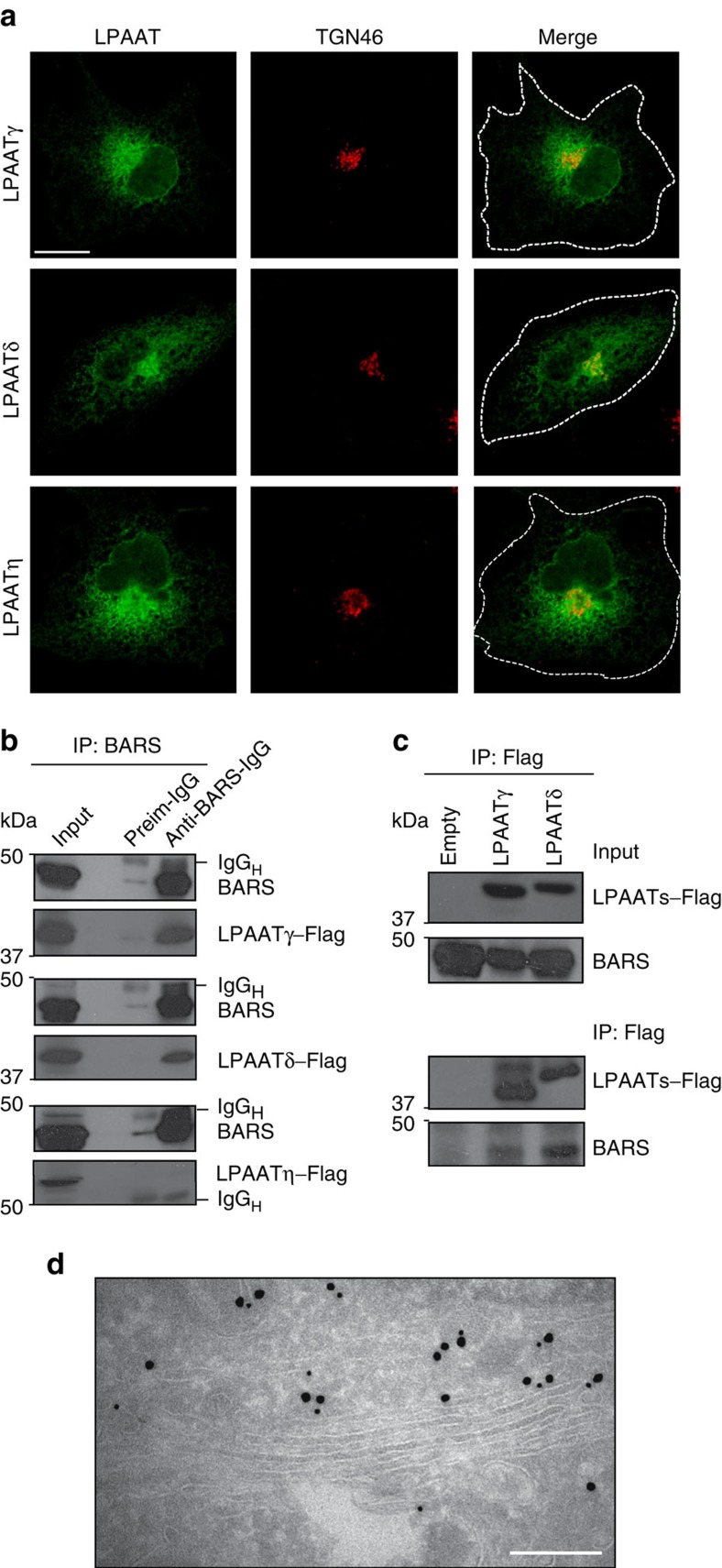
BARS interacts with LPAATδ. (**a**) Representative confocal microscopy images of COS7 cells transfected with Flag-tagged LPAATγ, LPAATδ and LPAATη, and fixed and processed for immunofluorescence with a monoclonal anti-Flag antibody (green) and with a polyclonal anti-TGN46 antibody (red; as indicated). Dotted lines indicate cell borders. (**b**) BARS immunoprecipitation (IP:BARS) of lysate from HeLa cells co-expressing BARS and LPAATγ–Flag, LPAATδ–Flag or LPAATη–Flag. Representative western blotting (antibodies as indicated) of total lysate (input) and immunoprecipitated proteins with preimmune-IgG (Preim-IgG) or anti-BARS-IgG (as indicated). IgG_H_, IgG heavy chain. (**c**) Immunoprecipitation with an anti-Flag antibody (IP:Flag) of lysate from HeLa cells co-transfected with BARS and LPAATγ–Flag, LPAATδ–Flag or the empty vector. Representative western blotting of total lysate (input) and Flag-immunoprecipitated proteins with an anti-Flag monoclonal antibody or the anti-BARS polyclonal antibody (as indicated). (**d**) Representative electron microscopy image of HeLa cells transfected with Flag-tagged LPAATδ for 24 h, and fixed and processed for cryo-immuno-electron microscopy with a monoclonal anti-Golgin-97 antibody (15-nm gold particles) and with a polyclonal anti-LPAATδ antibody (10-nm gold particles). Molecular weight standards (kDa) in **b** and **c**, are indicated on the left of each panel. Data are representative of three independent experiments. Scale bars, 10 μm (**a**); 200 nm (**d**).

**Figure 2:**
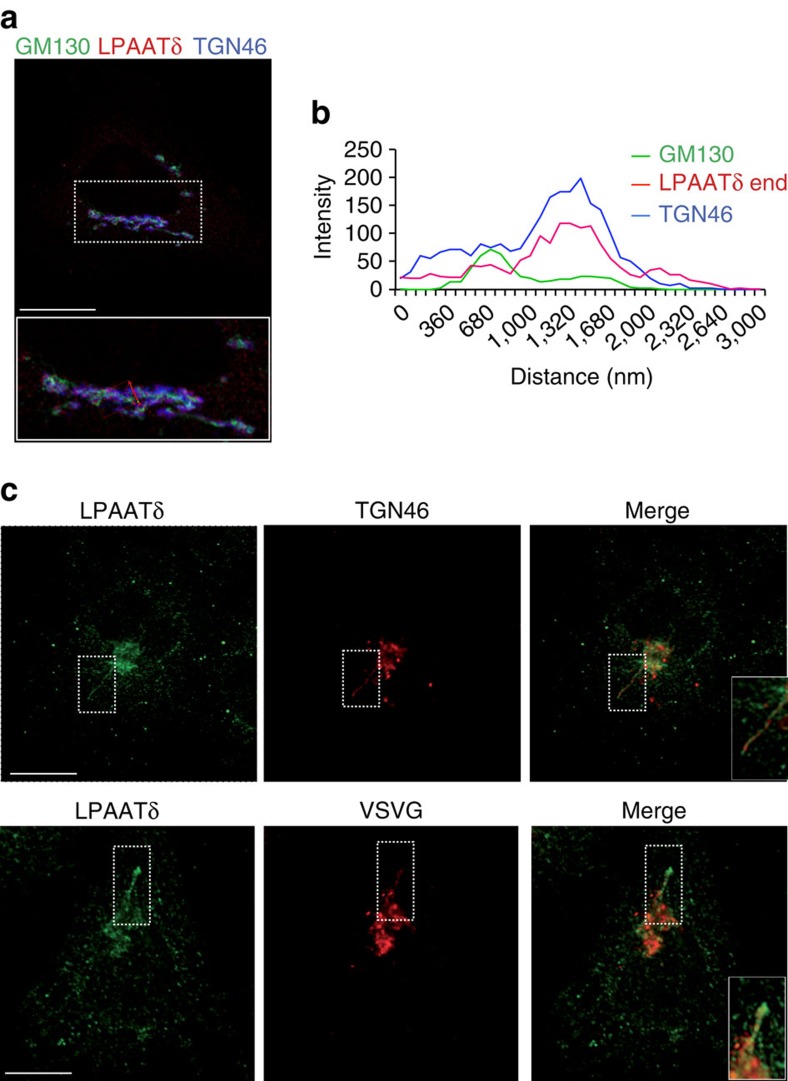
LPAATδ is a *trans*-Golgi–localized enzyme. (**a**) Representative confocal microscopy image of HeLa cells at steady-state fixed and labelled with a polyclonal anti-LPAATδ antibody (endogenous LPAATδ; red), with a monoclonal anti-GM130 antibody (green) and with an anti-TGN46 antibody (blue). Inset, bottom: Magnification of Golgi area. (**b**) Line scan across the Golgi area (red line across the magnified image in **a**) indicates the colocalization of LPAATδ with TGN46. (**c**) Representative confocal microscopy images of COS7 cells at steady-state (top) or VSV-infected and subjected to the VSVG TGN-exit assay (bottom). Cells were fixed and labelled with a polyclonal anti-LPAATδ antibody (endogenous LPAATδ; green) and with an anti-TGN46 antibody (red; top) or a monoclonal anti-VSVG antibody (red; bottom). Inset, right: magnification of tubular carrier precursors. Scale bars, 10 μm (**a**,**c**).

To examine whether BARS interacts with an LPAAT, we first sought to identify the LPAATs that localize to the Golgi, as most of the BARS-dependent fission reactions occur in this organelle^15^,^16^. We Flag-tagged and expressed the available mammalian LPAATs and inspected their localization by immunofluorescence microscopy. LPAATγ, LPAATδ and LPAATη localized to both the Golgi and the endoplasmic reticulum (ER) (Fig. 1a), while LPAATβ and LPAATɛ localized to the ER and mitochondria, respectively (Supplementary Fig. 1; see also refs 30, 31). We thus examined whether BARS interacts with the Golgi LPAATs by co-expressing BARS with each of these transferases and testing for co-immunoprecipitation of the two proteins. BARS co-precipitated with LPAATγ and LPAATδ (and vice versa), but not with LPAATη (Fig. 1b,c). LPAATγ has been shown to reside at the *cis*-Golgi and regulate Golgi structure and retrograde transport to the ER^33^,^34^, while LPAATδ has no known Golgi-related function to date. We asked whether the LPAATδ location might be compatible with a role in post-Golgi traffic by immuno-electron microscopy of the Flag-tagged protein (Fig. 1d). Tagged LPAATδ localized preferentially in the *trans*-Golgi and in the TGN (Fig. 1d), and to a lesser extent in the ER in the cell periphery. We also examined the distribution of the endogenous protein using specific antibodies and immunofluorescence microscopy (Fig. 2). The endogenous LPAATδ showed a localization that was very similar but not identical to that of the tagged protein, in that it was visualized mostly in the elongating tubules emanating from the TGN (Fig. 2), and gave a weaker signal in the cell periphery compared with the tagged protein. Of note, while this manuscript was being revised, it was reported that LPAATδ localizes to the mitochondrial outer membrane in murine cells^35^. This differs from our findings that LPAATδ localizes in the Golgi complex. However, cases of dual localization of the same transmembrane protein to the secretory pathway and to outer mitochondrial membrane have been reported several times^36^,^37^,^38^ and the mechanism of this dual localization is partially understood^39^. We thus considered that LPAATδ might localize both to the Golgi and the mitochondria, but that only the Golgi LPAATδ can be stained using the low titre antibody conditions optimized for specificity in our study, while LPAATδ in mitochondria might require a higher antibody titre for detection. We find that this is indeed the case, in all of the cell types tested (Supplementary Fig. 2a,b). Moreover, both the Golgi and mitochondrial signals were abolished by LPAATδ depletion, that is, are specific (Supplementary Fig. 2c). We conclude that LPAATδ can localize both in the secretory pathway and to a lower extent in mitochondria in most or all cells, though we do not exclude that the LPAATδ distribution might be partially cell type-dependent.

**Figure 3:**
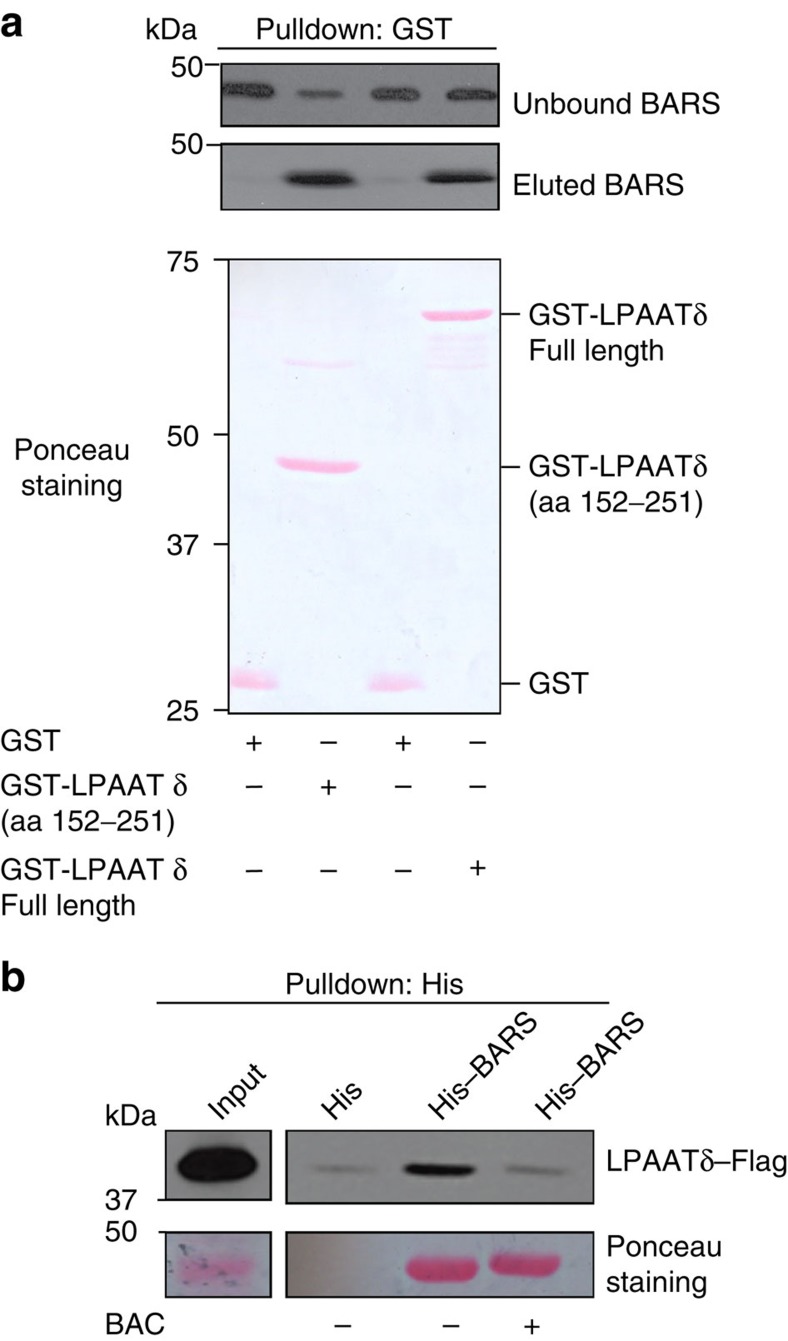
BARS binds LPAATδ directly. (**a**) Representative GST pull-down of equimolar amounts of GST, GST-LPAATδ (aa 152–251) and GST-LPAATδ (full length) purified recombinant proteins, for His-BARS, with unbound and eluted proteins analysed by western blotting (top, anti-BARS monoclonal antibody). GST fusion proteins were revealed by Ponceau staining (bottom). (**b**) Representative histidine pull-down for His or His-BARS beads of lysates from COS7 cells transfected with LPAATδ–Flag. Beads were treated with buffer alone (−) or with HPLC-purified BAC (BAC+), and then incubated with the lysates. The eluted proteins were analysed by western blotting using a monoclonal anti-Flag antibody (top), with the pulled down His-BARS revealed by Ponceau staining (bottom). Molecular weight standards (kDa) are indicated on the left of each panel. Data are representative of three independent experiments.

We then asked whether BARS binds directly to LPAATδ by performing pull-down experiments with the two purified recombinant proteins. After coincubation, recombinant BARS and LPAATδ (prepared as described in Methods) co-precipitated efficiently (Fig. 3a). We also tested for binding between BARS and the 152–251aa portion of LPAATδ, a cytosolic segment exposed at the protein surface (see models in ref. 30). BARS co-precipitated also with this fragment (Fig. 3a). These data indicate direct binding between the two proteins.

Finally, we examined whether LPAATδ binds selectively with the monomeric fission-competent form of BARS^15^,^16^. As noted, BARS shifts between monomeric and dimeric conformations depending on ligand binding to its Rossman fold. Dimerization is promoted by the binding either to NAD(H)^19^,^26^,^27^,^28^ or to the Brefeldin A–ADP-ribosylated conjugate (BAC), an ADP-ribosylated metabolite of brefeldin A, which locks BARS in the dimeric fission-inactive conformation^28^. Either BARS alone or BAC-bound BARS were added to lysates from cells overexpressing LPAATδ. BARS and LPAATδ interacted efficiently as judged from co-precipitation (see above), as expected, and this interaction was markedly reduced by the binding of BARS with BAC (Fig. 3b) (NAD(H) had similar effects, not shown), indicating that LPAATδ preferentially binds monomeric BARS.

We conclude that the LPAATδ is located in the elongating tubules emanating from the TGN (Fig. 2) and binds directly and selectively the monomeric fission-active form of BARS (Fig. 3).

### Bacterial LPAAT competes with LPAATδ for BARS binding

As noted, recombinant BARS purified from *Escherichia coli* is associated with low levels of LPAAT activity. Since this activity belongs to the bacterial enzyme^5^,^29^ (see also Supplementary Fig. 3), we carried out pull-down experiments to examine whether BARS and *E. coli* LPAAT might bind to each other and do so in a specific manner. His-tagged *E. coli* LPAAT (which can be prepared in soluble form)^40^ showed strong binding with BARS (Supplementary Fig. 4a), and this binding was abolished by pre-treatment of BARS with BAC or NAD(H) (Supplementary Fig. 4b,c). This indicates that, like the mammalian enzyme (Fig. 3b), the *E. coli* LPAAT binds selectively to monomeric BARS (Supplementary Fig. 4b,c). We also asked whether *E. coli* LPAAT competes with mammalian LPAATδ for binding to BARS. This was the case (though the effect was partial; Supplementary Fig. 4d), suggesting that the mammalian and bacterial LPAATs bind to the same BARS domain, and that the BARS-binding surfaces of the mammalian LPAATδ and *E. coli* LPAAT are conserved. Considering the evolutionary distance between the two LPAATs, this is somewhat surprising. It is conceivable that the evolutionary ancestors of LPAATδ and BARS (probably an LPAAT and a D-hydroxyacid dehydrogenase, respectively)^40^, which are fundamental metabolic enzymes, were interactors in an ancient metabolic multi-enzyme complex^41^, and that this interaction was maintained through evolution in different functional contexts.

Regardless, these data explain our previous observations that recombinant BARS from *E. coli* is associated with an LPAAT activity^5^, which, as we now know, belongs to the *E. coli* enzyme. They also provides a potentially useful tool (the soluble bacterial enzyme) for *in vitro* reconstitution of BARS-dependent fission.

### LPAATδ is required for post-Golgi carrier fission

**Figure 4:**
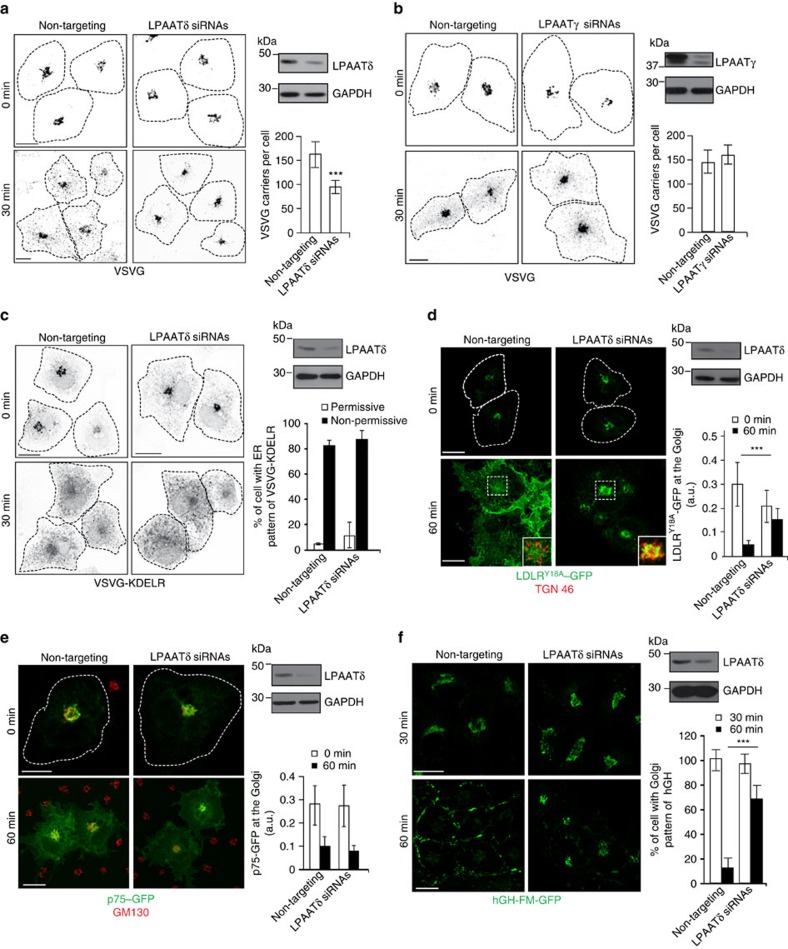
LPAATδ is required for the fission of basolaterally-directed carriers. (**a**,**b**) Representative images of COS7 cells treated with non-targeting and *LPAATδ* siRNAs in **a** or *LPAATγ* siRNAs in **b** before VSV infection and the TGN-exit assay with 0.5% tannic acid. The cells were fixed following a 20 °C block (0 min) or 30 min after the shift to 32 °C, and stained for VSVG-positive post-Golgi carriers. Quantification is on the right (see Methods). (**c**–**e**) Representative images of COS7 cells treated with non-targeting and *LPAATδ* siRNAs before co-transfection for the last 16 h with VSVG-ts045-KDELR-myc in **c** or with the endocytosis-defective LDLR–GFP receptor in **d** or with a plasmid encoding p75–GFP in **e**. (**c**) The distribution of the chimeric KDELR was examined following the shift to the non-permissive temperature for 30 min. Quantification of ER distribution of the chimeric KDELR (right). (**d**) Following the 2 h at 20 °C transport block (0 min) and 60 min after the shift to 32 °C (with cycloheximide), the cells were fixed and labelled with TGN46 (Golgi marker; red). Insets: enlarged view of merged signals for the Golgi area. (**e**) Following the 3 h at 20 °C block (0 min) and 60 min after the shift to 32 °C (with cycloheximide), the cells were fixed and stained for GM130 (Golgi marker; red). (**d**,**e**) Quantification of LDLR^Y18A^–GFP in **d** and p75–GFP in **e** in the Golgi area (right). (**f**) Representative microscopy of HeLa cells stably transfected with hGH-FM–GFP and treated with non-targeting and *LPAATδ* siRNAs before subjection to a secretion assay. Release of hGH-FM–GFP from ER was performed by the addition of DD-solubilizer at 37 °C for the indicated times. Quantification of hGH-FM–GFP in the Golgi area (right). (**a**–**e**), Dotted lines indicate cell borders. (**a**–**f**), The efficiency of interference was monitored by western blotting of the cell lysates using polyclonal anti-LPAATδ in **a**,**c**–**f**, or polyclonal anti-LPAATγ in **b** antibodies. Glyceraldehyde 3-phosphate dehydrogenase (GAPDH) is shown for the internal protein levels and molecular weight standards (kDa) are indicated on the left of each panel in **a**–**f**. Data are means±s.d. of three independent experiments. ***P<0.005 (Student's *t*-tests). Scale bars, 10 μm.

The TGN localization and the interaction of LPAATδ with BARS persuaded us to investigate the role of this LPAAT isoform in the BARS-dependent formation of post-Golgi carriers^14^. LPAATδ was silenced with specific small-interfering RNAs (siRNAs), and the formation of carriers, as well as the rate of transport to the plasma membrane was monitored using the temperature-sensitive vesicular stomatitis virus G protein (ts045-VSVG; henceforth, VSVG)^42^ as a traffic marker. The exit of VSVG out of the Golgi can be synchronized by accumulating VSVG in the TGN at 20 °C and then shifting the temperature to 32 °C^14^. The formation of VSVG-containing carriers from the TGN was visualized by immunofluorescence microscopy and quantified by inhibiting the fusion of these carriers with the plasma membrane with tannic acid^14^,^15^,^43^, which results in accumulating carriers close to the cell surface. The depletion of LPAATδ markedly reduced the formation of VSVG-containing carriers (Fig. 4a), whereas the depletion of LPAATγ, which is located at the *cis*-Golgi and is involved in Golgi-to-ER traffic^33^,^34^, had no effect (Fig. 4b). LPAATδ depletion had no effect on cell viability, growth and morphology for the duration of the experiment and longer.

To determine whether the reduction in VSVG-containing carriers was due to inhibition of carrier budding or of fission, we monitored carrier formation in living cells expressing VSVG–green fluorescent protein (VSVG–GFP). While control cells exhibited dynamic tubules that formed and underwent multiple fission events to generate a large number of free-moving carriers, which then fused with the plasma membrane, LPAATδ-depleted cells showed several long (>10 μm) tubular extensions that contained VSVG–GFP. These tubules represent carrier precursors that elongate out of the Golgi but do not detach to form mature transport intermediates^14^,^15^ (Supplementary Movies 1 and 2). When they (rarely) did detach, however, they could be seen to move towards, and fuse with, the plasma membrane (Supplementary Movie 2). This phenotype was similar to that induced by expressing BARS dominant-negative mutants or by depleting BARS^14^,^15^ or by depleting 14-3-3γ, a key component of the PI4KIIIβ–14-3-3γ dimer–BARS complex required for fission, or by expressing dominant-negative mutants of PKD^16^. Very similar effects were induced by microinjection of an affinity-purified antibody against LPAATδ (Supplementary Movie 3; see also Supplementary Fig. 5) and by the general LPAAT inhibitor CI-976 (ref. 44; Supplementary Movie 4).

These findings indicate an essential role for LPAATδ in the fission of tubular carriers exiting the Golgi complex.

We also determined the role of LPAATδ in other traffic steps. We first examined retrograde traffic from the Golgi to the ER (which is known to require LPAATγ)^33^,^34^, by monitoring the well-characterized retrograde transport marker VSVG-KDELR (a fusion of VSVG with the KDEL receptor)^45^. The transport of VSVG-KDELR was not affected by LPAATδ depletion (Fig. 4c). Second, since BARS controls the fission of basolateral but not apical carriers^14^, we examined the role of LPAATδ in Golgi export of the apical cargo p75 (ref. 46) in comparison with export of the LDL receptor (a basolateral cargo, like VSVG)^47^. Depletion of LPAATδ inhibited export of the LDL receptor (Fig. 4d), but not that of p75 (Fig. 4e). Finally, we probed a soluble basolateral cargo, the stably expressed constitutively secreted GFP-tagged variant of the human growth hormone (hGH)^48^. Depletion of LPAATδ strongly inhibited export of hGH-FM–GFP from the Golgi to the plasma membrane (Fig. 4f). Therefore, like BARS, LPAATδ appears to be selectively required for the fission of basolateral carriers.

### The LPAATδ activity is needed for post-Golgi carrier fission

**Figure 5:**
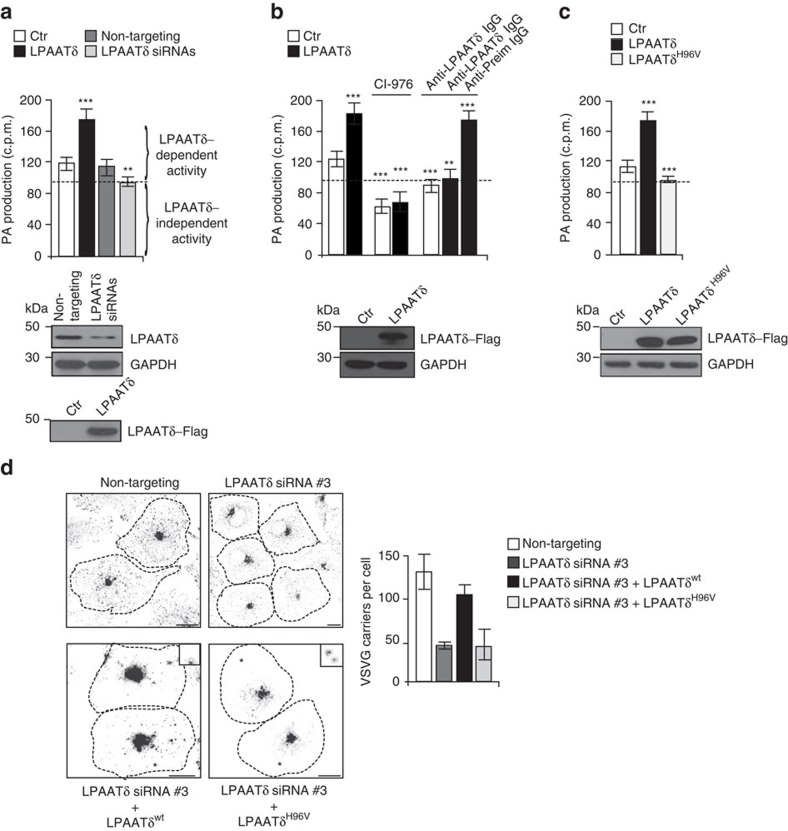
LPAATδ is a canonical LPAAT and its activity is required for post-Golgi carrier formation. (**a**) Quantification of phosphatidic acid (PA) production in the LPAAT assay for post-nuclear supernatants from HeLa cells transfected for 48 h with an empty Flag-vector (Ctr) or with LPAATδ–Flag (LPAATδ), or for 72 h with non-targeting or *LPAATδ* siRNAs. The curly brackets indicate the LPAATδ-dependent and independent activities, as defined in Methods. (**b**) Quantification as in **a**, with the post-nuclear fractions also incubated with 50 μM CI-976, or polyclonal anti-LPAATδ antibody (anti-LPAATδ IgG), or anti-preimmune-IgG (anti-Preim-IgG; as control) for 30 min at 25 °C before LPAAT assay. (**c**) Quantification as in **a**, also in parallel with the LPAATδ^H96V^–Flag (LPAATδ^H96V^) catalytically inactive mutant. (**a**–**c**) The dashed line indicates the level of endogenous LPAAT activity not associated with LPAATδ (see text for details). Bottom: representative western blotting with an anti-Flag antibody, for the transfection efficiencies of these proteins used for the LPAAT assays. Glyceraldehyde 3-phosphate dehydrogenase (GAPDH) is shown for the internal protein levels. Molecular weight standards (kDa) in **a**–**c**, are indicated on the left of each panel. (**d**) Representative images of COS7 cells transfected with non-targeting or *LPAATδ* siRNA (duplex #3; *LPAATδ* siRNA #3), and with LPAATδ^wt^–Flag or the LPAATδ^H96V^–Flag catalytically inactive mutant, and subjected to VSV infection and the TGN-exit assay with 0.5% tannic acid. The cells were fixed 30 min after the shift to the permissive temperature (32 °C) and processed for immunofluorescence with monoclonal anti-Flag and polyclonal anti-VSVG (p5D4) antibodies, to monitor formation of VSVG-containing carriers. Dotted lines show cell borders. Asterisks represent LPAATδ^wt^–Flag and LPAATδ^H96V^–Flag transfected cells (see inserts for staining with anti-Flag antibody; bottom images). Scale bars, 10 μm. Quantification of VSVG-positive carriers (right). Data are means±s.d. of three independent experiments. **P<0.01, ***P<0.005 versus control (Student's *t*-tests).

To examine whether the enzymatic activity of LPAATδ^49^ is required for fission, we first set-up an assay to determine such activity. We prepared and incubated post-nuclear supernatants with the acyl donor [1-^14^C]-oleoyl-CoA and the acyl acceptor oleoyl-LPA, with [1-^14^C]-PA measured as the reaction product (see Methods and Fig. 5a). These are standard conditions used for LPAAT assays^49^,^50^, as attempts to purify the LPAAT enzymes result in activity loss^49^,^50^. Extracts from control cells showed an efficient LPAAT activity, which was suppressed by the general LPAAT inhibitor CI-976 (Fig. 5b)^34^,^51^. A difficulty with these extracts is that they contain multiple LPAATs. We therefore designed conditions to selectively determine the LPAATδ activity (Fig. 5a), based on suppressing or overexpressing this enzyme. Extracts from LPAATδ-depleted cells (Fig. 5a) or treatment of control extracts with a specific affinity-purified antibody against LPAATδ (Fig. 5b) (see Methods for antibody characterization) showed a reproducibly lower (≈25%) activity than in controls (Fig. 5a,b), confirming that LPAATδ is responsible for a fraction of the total LPAAT activity, and in line with the presence of other LPAATs such as the abundant glycerolipid synthetic enzymes LPAATα and LPAATβ^30^,^52^. Extracts from LPAATδ-overexpressing cells showed a ≈40% increase in LPAAT activity over controls (Fig. 5a–c), and this increase was completely inhibited by antibodies against LPAATδ (Fig. 5b), down to the levels found in the absence of LPAATδ (Fig. 5a).

LPAATδ silencing or overexpression did not affect the cellular levels of other LPAATs (Supplementary Fig. 6). Similar data were obtained using [1-^14^C]-palmitoyl-CoA as acyl donor and arachidonoyl-LPA as acyl acceptor (extracts from LPAATδ-overexpressing cells showed a ≈45% increase in LPAAT activity over controls). We thus define the LPAATδ-dependent activity (or LPAATδ activity) as the activity value of LPAATδ-overexpressing extracts (as measured using concentrations of substrates below the *K*_m_ values, see below) minus the activity value of LPAATδ-depleted (or antibody-treated) extracts (Fig. 5a,b; see dashed line and Methods). The maximum reaction rate *V*_max_ and Michaelis–Menten constant *K*_m_ of this LPAATδ activity were 38±3 nmol min^−1^ per mg^2^ protein and 58±18 μM, respectively, for oleoyl-CoA, and 38±1 nmol min^−1^ per mg^2^ protein and 29±1 μM, respectively, for oleoyl-LPA (data are means±s.d. of three independent experiments). These rates are comparable to those reported for LPAATγ^53^. Importantly, they are potentially sufficient, depending on substrate availability, to change the PA concentrations in the TGN rapidly and substantially.

Finally, we asked whether the LPAATδ catalytic activity is required for post-Golgi carrier fission. We generated a single-point mutant (LPAATδ^H96V^) in the conserved acyltransferase catalytic site of LPAATδ (NHX_4_D)^30^,^54^. Overexpressed LPAATδ^H96V^ was devoid of LPAAT activity (Fig. 5c), confirming that LPAATδ is a canonical LPAAT family member^30^,^54^. We then depleted cells of LPAATδ, with the consequent inhibition of the post-Golgi transport of VSVG (see Fig. 4a and Supplementary Movie 2) and expressed either a siRNA-resistant variant of LPAATδ or of the catalytically dead LPAATδ^H96V^ mutant. Only the wild-type LPAATδ rescued carrier formation, while LPAATδ^H96V^ was completely inactive (Fig. 5d). These data indicate that the catalytic activity of LPAATδ, and hence most likely the formation of PA from LPA, is necessary for post-Golgi carrier formation.

### Activation of LPAATδ by BARS is required for carrier fission

**Figure 6:**
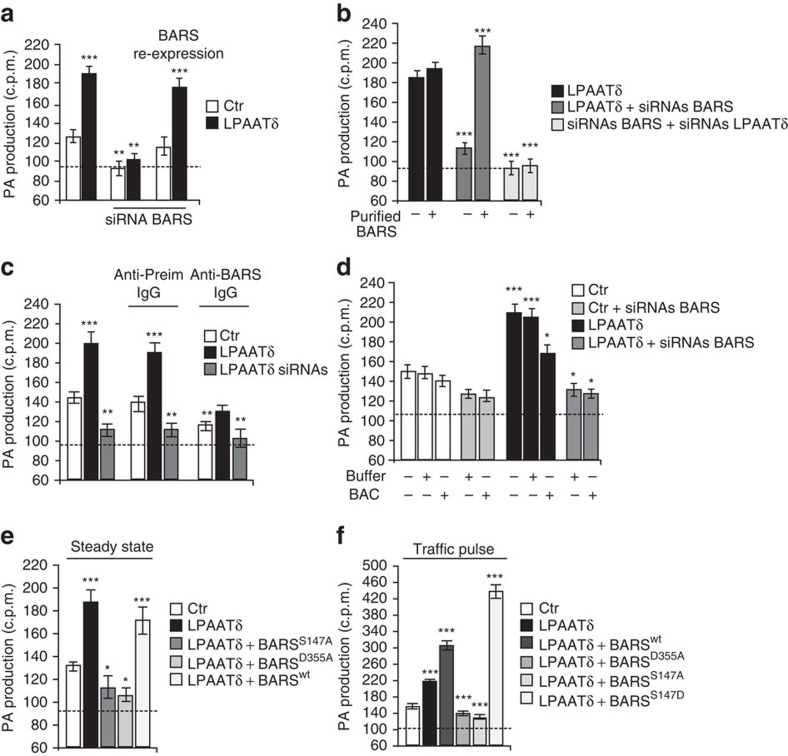
BARS activates LPAATδ and this activation is required for post-Golgi carrier formation. Quantification of phosphatidic acid (PA) production in the LPAAT assay for post-nuclear supernatants from HeLa cells transfected with: (**a**) empty Flag-vector (Ctr) or LPAATδ–Flag (LPAATδ) and with *BARS* siRNAs for 48 h, and with the last 12 h with siRNA-resistant replacement BARS–YFP-encoding vector (BARS re-expression); (**b**) LPAATδ–Flag (LPAATδ) and/or with *BARS* siRNAs and/or *LPAATδ* siRNAs. Post-nuclear fractions were incubated with immunopurified BARS (Purified BARS) for 30 min at 25 °C before LPAAT assay (as indicated). (**c**) Quantification of PA production in the LPAAT assay for post-nuclear supernatants from HeLa cells transfected with empty Flag-vector (Ctr) or LPAATδ–Flag (LPAATδ) for 48 h or with *LPAATδ* siRNAs for 72 h. The anti-BARS polyclonal antibody (Anti-BARS IgG) or anti-preimmune-IgG (Anti-Preim IgG, as control) was incubated with the indicated post-nuclear fraction for 30 min at 25 °C before LPAAT assay. (**d**) Quantification of PA production in the LPAAT assay for post-nuclear supernatants from HeLa cells transfected with empty Flag-vector (Ctr) or LPAATδ–Flag (LPAATδ) and with *BARS* siRNAs for 48 h. Post-nuclear fractions were incubated with HPLC-purified BAC (BAC+) or with buffer alone (Buffer+) for 30 min at 25 °C before LPAAT assay (as indicated). (**e**,**f**) Quantification of PA production in the LPAAT assay for post-nuclear supernatants from HeLa cells transfected with: (**e**) empty Flag-vector (Ctr) or LPAATδ–Flag (LPAATδ) for 48 h and the last 12 h with BARS^S147A^–YFP, BARS^D355A^–YFP or BARS^wt^–YFP (as indicated); (**f**) empty Flag-vector (Ctr) or LPAATδ–Flag (LPAATδ) for 48 h and the last 12 h with BARS^wt^–YFP, BARS^D355A^–YFP, BARS^S147A^–YFP or BARS^S147D^–YFP (as indicated). Cells were infected with VSV, subjected to TGN-exit assay and post-nuclear fractionations were prepared 10 min after the shift to 32 °C temperature-release block. The dashed line indicates the level of endogenous LPAAT activity not associated with LPAATδ (see text for details). Data are means±s.d. of three independent experiments. *P<0.05, **P<0.01, ***P<0.005 versus control (Student's *t*-test). See also Supplementary Fig. 7 for LPAATδ and BARS (wild type and mutants) expression levels in post-nuclear samples.

Since BARS and LPAATδ interact directly (Fig. 3a) and are required for post-Golgi carrier fission (see refs 14, 15, and Supplementary Movies 2 and 3, respectively), we asked whether BARS regulates the enzymatic activity of LPAATδ, and whether this regulation is required for carrier fission. We first silenced BARS and measured the LPAATδ activity in cell extracts. BARS depletion abolished the LPAATδ activity (Fig. 6a). We then re-expressed BARS in BARS-silenced cells, using a siRNA-resistant replacement BARS construct (Supplementary Fig. 7a). This nearly completely restored the LPAATδ activity (Fig. 6a). As a specificity control, the above BARS manipulations did not affect the cellular levels of LPAATδ (Supplementary Fig. 7a) or of other LPAATs (Supplementary Fig. 7b). These results indicate that LPAATδ requires BARS to express its activity.

We then sought to manipulate the BARS levels acutely in *in vitro* lysates to exclude transcriptional or compensatory effects that might arise in siRNA-depletion experiments^16^,^24^. We prepared extracts from LPAATδ-expressing and BARS-depleted cells, where LPAATδ is inactive (Fig. 6a), and added immunopurified BARS to the assay mixture to a final BARS concentration of 5 μg ml^−1^ (comparable to the levels of endogenous BARS)^14^. Under these conditions, BARS completely restored the LPAATδ-dependent activity (Fig. 6b) (of note, the LPAAT activity associated with immunopurified BARS was quantitatively negligible)^5^,^29^. We also added BARS to control LPAATδ-expressing extracts. This treatment only slightly stimulated the LPAATδ-dependent activity (Fig. 6b), suggesting that endogenous BARS is sufficient to activate LPAATδ, at least in extracts from quiescent cells (that is, in cells not subjected to a traffic pulse; see below). As a further control, we used extracts from cells depleted of both BARS and LPAATδ (Fig. 6b). Here addition of immunopurified BARS had no effect on the LPAAT activity, suggesting that other LPAAT isoforms are not detectably stimulated by BARS, at least under our experimental conditions.

Further along this line, we sought to inhibit BARS by adding to the LPAAT assay mixture a characterized neutralizing affinity-purified anti-BARS antibody which, when microinjected into cells, inhibits carrier fission^14^,^15^,^55^. This antibody inhibited the LPAATδ-dependent activity, while preimmune-IgG addition had no effect (Fig. 6c). Moreover, a pre-treatment of the assay mixture with BAC, which locks BARS in its dimeric fission-incompetent conformation and inhibits the BARS-LPAATδ binding (see above and Fig. 3b)^28^, reduced the LPAATδ activity (Fig. 6d), supporting the role of BARS in LPAATδ activation and indicating that monomeric BARS is required for LPAATδ to express its enzymatic activity.

We further examined the relationship between LPAATδ activity, BARS and carrier fission by expressing suitable BARS mutants. Previously, we have characterized two single-point mutants, BARS^D355A^ and BARS^S147A^ that have dominant-negative effects on carrier fission in living cells^14^,^15^. We tested these mutants in the LPAATδ activity assay by co-expressing each of them with LPAATδ. Both nearly completely inhibited the LPAATδ-dependent activity (Fig. 6e), again without affecting LPAATδ expression levels (Supplementary Fig. 7c). As a control, we tested the effects of overexpressing wild-type BARS (of note, wild-type BARS and the dominant-negative mutants showed comparable expression levels in these experiments; see Supplementary Fig. 7c). Overexpressed BARS did not have significant effects on the LPAATδ activity in extracts from ‘quiescent' cells (Fig. 6e), again suggesting that basal BARS levels are sufficient to support LPAATδ activity under these conditions (see above). We noted, however, that BARS is recruited to the Golgi during a traffic pulse, which suggests that active traffic increases the requirement for BARS^15^, possibly to stimulate LPAATδ. Indeed, in extracts prepared during a traffic pulse, the overexpression of BARS stimulated the LPAATδ activity over control levels (Fig. 6f). Moreover, in these extracts, the expression of the fission-active BARS^S147D^ mutant that mimics the activatory phosphorylation of BARS on Ser147^15^,^17^,^18^ stimulated the LPAATδ activity to an even greater extent (Fig. 6f). These collective data are consistent with the idea that LPAATδ is activated during a traffic pulse and that this activation requires higher (local) levels of BARS than those present in quiescent cells. Testing this possibility further, we examined whether the interaction between BARS and LPAATδ might be enhanced during a pulse. Co-precipitation experiments indicate that BARS co-precipitates more efficiently with LPAATδ under pulse conditions (Supplementary Fig. 8).

**Figure 7:**
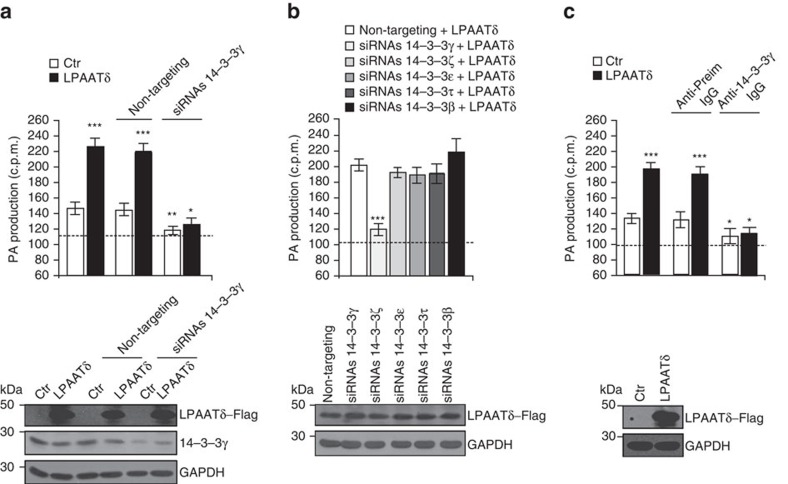
14-3-3γ but not other 14-3-3 isoforms is required for LPAATδ activity. (**a**–**c**) Quantification of phosphatidic acid (PA) production in the LPAAT assay for post-nuclear supernatants from HeLa cells transfected with the empty Flag-vector (Ctr) or LPAATδ–Flag (LPAATδ) plus: (**a**) transfection with non-targeting siRNAs or *14-3-3γ* siRNAs for 48 h (as indicated); (**b**) transfection with *14-3-3γ, ζ, ɛ, τ* and *β* siRNAs for 48 h (as indicated); (**c**) treatment of the post-nuclear supernatant with an anti-14-3-3γ polyclonal antibody (Anti-14-3-3γ IgG) or anti-preimmune-IgG (Anti-Preim IgG, as control) for 30 min at 25 °C before the LPAAT assay. (**a**–**c**) The dashed line indicates the level of endogenous LPAAT activity not associated with LPAATδ (see text for details). Bottom: representative western blotting with an anti-Flag antibody in **a**–**c**, and with an anti-14-3-3γ monoclonal antibody in **a** to monitor the transfection of LPAATδ and the depletion of 14-3-3γ in the lysate used for LPAAT assay. Glyceraldehyde 3-phosphate dehydrogenase (GAPDH) is shown for the internal protein levels and molecular weight standards (kDa) are indicated on the left of each panel. Data are means±s.d. of three independent experiments. *P<0.05; **P<0.01; ***P<0.005 versus control (Student's *t*-tests). See also Supplementary Fig. 9 for 14-3-3s expression levels.

We also tested the role of the PI4KIIIβ–14-3-3γ dimer–BARS complex in LPAATδ activation. As noted, within this complex, 14-3-3γ binds to phosphorylated Ser147 in the BARS dimerization interface and is necessary for Golgi carrier fission^15^,^16^. The LPAATδ activity of cell extracts was markedly suppressed by 14-3-3γ depletion (Fig. 7a), while depletion of other 14-3-3 isoforms had no effect (Fig. 7b and see also Supplementary Fig. 9). Moreover, addition to cell extracts of a characterized affinity-purified anti-14-3-3γ antibody^15^ also suppressed the LPAATδ activity (Fig. 7c). These data indicate that 14-3-3γ is required for LPAATδ activity, presumably because it stabilizes BARS in its monomeric fission-competent conformation.

Finally, we repeated a few of the above experiments using a Golgi-membrane-enriched fraction (Supplementary Fig. 10a). Here the LPAATδ overexpression increased LPAAT activity by ≈60% over controls (Supplementary Fig. 10b), while LPAATδ-depletion or treatment of the membranes with the anti-LPAATδ antibody (see also Fig. 5a,b) reduced the activity by ≈35% compared with controls (Supplementary Fig. 10b,c), in line with the notion that Golgi membranes are enriched in LPAATδ over total cell extracts (compare with Fig. 5a). Also, as seen with total extracts (Fig. 6), the LPAATδ activity in Golgi membranes was nearly abolished by BARS depletion or inhibition by the anti-BARS antibody (Supplementary Fig. 10d).

In summary, a number of experimental results based on BARS silencing or overexpression, or on the use of BARS activatory or dominant-negative mutants and of anti-BARS antibodies and inhibitors, or on manipulations of the BARS-containing complex, converge towards the conclusion that the BARS-induced LPAATδ activation controls the fission of Golgi carriers. The stimulation of LPAATδ by BARS is potent, and appears to occur rapidly, most likely via the direct physical interaction between BARS and LPAATδ during assembly of the BARS protein complex that is required for carrier formation.

### The BARS–LPAATδ interaction occurs at the Golgi complex

**Figure 8:**
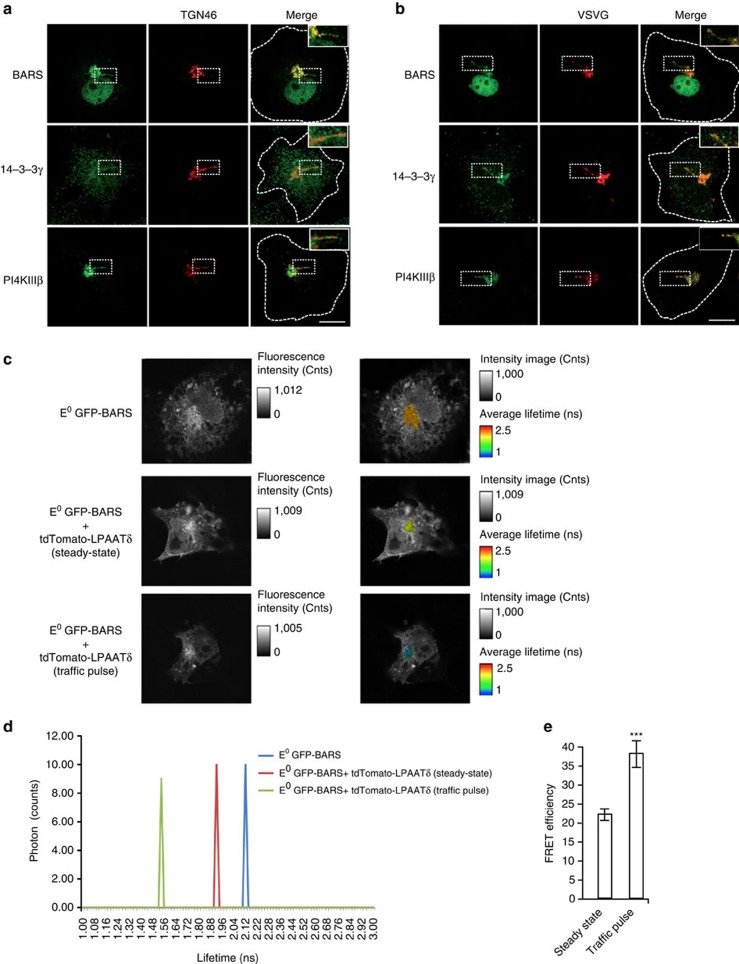
BARS colocalizes with 14-3-3γ and PI4KIIIβ at the TGN and in carrier precursors and interacts with LPAATδ at the Golgi. (**a**,**b**) Representative images of COS7 cells at steady state in **a**, or VSV-infected and subjected to the VSVG TGN-exit assay in **b**. The cells were fixed and labelled with polyclonal anti-BARS, anti-14-3-3γ or anti-PI4KIIIβ (**a**,**b**; green) antibodies, and with an anti-TGN46 antibody (**a**; red) or a monoclonal anti-VSVG antibody (**b**; red). Insets, right: magnification of the tubular carrier precursors in the Golgi area. Dotted lines indicate cell borders. Scale bars, 10 μm. (**c**,**d**) Pixel-by-pixel FLIM analysis and photon distributions of COS7 cells transfected with E^0^GFP–BARS alone or in combination with tdTomato-LPAATδ and fixed at the steady state or during the traffic pulse (as indicated). (**c**) The shortening of E^0^GFP–BARS donor lifetime in the presence of the tdTomato-LPAATδ acceptor due to FRET is lower in the steady-state fixed cell (where a basal interaction between the two proteins occurs) and is higher, as indicated by the blue pixels, in the traffic pulse cell (fixed after 10 min of the 32 °C temperature-release block, see Methods) where the interaction between the two proteins is quantitatively increased (see also Supplementary Fig. 8). (**d**) Representative photon-counting events during the time of the donor E^0^GFP–BARS fluorescence emission, measured in pixels containing >300 photons in the Golgi area, when overexpressed alone (blue) or with tdTomato-LPAATδ under steady-state (red) or during traffic pulse (green) conditions. The average fluorescence lifetime of E^0^GFP–BARS were 2.08±0.07 ns (E^0^GFP–BARS alone), and 1.62±0.22 ns and 1.28±0.27 ns (with tdTomato-LPAATδ) under steady-state or during traffic pulse conditions, respectively. (**e**) Quantification of FLIM-FRET efficiency in the Golgi area under steady-state or during a traffic pulse conditions (as indicated). Data are mean±s.d. (*n*=10 cells per condition). ***P<0.005 (Student's *t*-tests) versus steady state.

The effects of BARS and LPAATδ on the fission of carriers emanating from the Golgi suggest that this BARS–LPAATδ interaction occurs at this organelle. To verify this notion, we first re-examined the Golgi localization of BARS and of the other components of the complex, 14-3-3γ and PI4KIIIβ, focusing, in particular, on the carrier precursors elongating out of the Golgi, where fission takes place. Similar to LPAATδ (Fig. 2), these proteins were all seen to localize at the TGN (Fig. 8a) and on the VSVG-containing tubular carriers that form during synchronized exit from the TGN (Fig. 8b). Next, we directly examined whether the interaction between BARS and LPAATδ occurs at the Golgi using an approach based on Förster resonance energy transfer (FRET) and fluorescence lifetime imaging microscopy (FLIM). This technique reveals the co-presence of suitable donor and acceptor fluorophores within the same complex (that is, at a distance of ≤8 nm). We expressed E^0^GFP–BARS as the FRET donor, and tdTomato-LPAATδ as the acceptor, and the donor lifetime was measured to assess FRET under steady state and traffic pulse conditions. A comparison of the donor decay lifetime in control cells (cells not expressing the acceptor) and in cells co-expressing donor and acceptor showed in the latter case a marked reduction of donor lifetime at the Golgi (Fig. 8c,d), indicating that FRET, and hence interaction, between the fluorescent partners takes place (Fig. 8e). Moreover, the FRET signal at the Golgi markedly increased during a VSVG traffic pulse (Fig. 8c–e), consistent with the enhanced BARS–LPAATδ interaction observed during a pulse (Supplementary Fig. 8). These results indicate that LPAATδ is in complex with BARS *in vivo* at the Golgi and that it colocalizes at the TGN with BARS, 14-3-3γ and PI4KIIIβ, in agreement with the BARS–LPAATδ co-precipitation and interaction data (Figs 1 and 3 and Supplementary Fig. 8).

## Discussion

The main finding of this study is that BARS induces fission of post-Golgi basolateral carriers by interacting directly with, and activating, the enzyme LPAATδ, a member of the acyltransferase family, which localizes at the TGN and converts LPA into PA. This indicates that the LPA–PA conversion plays a role in BARS-dependent fission. Notably, the metabolism of PA has been implicated in various aspects of membrane dynamics by other groups, albeit generally based on indirect evidence^18^,^21^,^34^,^56^,^57^,^58^.

Based on current and previous results, we propose the following working model for basolateral carrier formation. ARF initiates the process by recruiting and activating PI4KIIIβ at the Golgi. This produces a local increase in phosphatidylinositol 4-phosphate (PtdIns4*P*)^59^, which supports the budding of tubular carrier precursors, most likely through recruitment of PtdIns4*P*-binding proteins^16^. Carrier precursor budding is assisted by phospholipase A_2_ (PLA_2_) via production of positively curved lysolipids, including LPA, which facilitate the bending of membranes into tubules^2^,^33^,^34^,^60^,^61^. Concomitantly, BARS assembles with 14-3-3γ and PI4KIIIβ into a complex where 14-3-3γ binds to the BARS dimerization surface, and thus keeps BARS in a monomeric fission-competent conformation^15^,^16^. BARS then activates LPAATδ at the TGN and on the elongating tubular carrier precursors, where the PLA_2_-generated LPA is converted into PA by LPAATδ, leading to carrier fission, in a process that might be aided by the property of BARS itself and ARF to insert into lipid membranes^21^,^62^.

LPA and PA have biophysical properties that may be relevant for fission. PA has a highly charged headgroup close to the glycerol backbone, a tendency to form intramolecular and intermolecular hydrogen bonds and segregate into microdomains. Because PA can act as a binding site for proteins bearing amphipathic/hydrophobic surfaces^56^, which can lead to fission through the shallow hydrophobic insertion mechanism^11^, its above properties might cause PA-binding proteins to insert into membranes at the optimal depth and extent for causing fission. Also of note is that BARS itself^21^ has been reported to bind PA. One can thus hypothesize that a runaway process might take place at the tubular precursor surface by which BARS-bound LPAATδ converts LPA into PA, causing more BARS molecules to be recruited to the membrane and bind to LPAATδ, and hence more LPA to be converted into PA (similar to the positive-feedback Sec7–Arf1 loop whereby Sec7 activates Arf1 to recruit more Arf1 to activate more Sec7 and so on)^63^, until the local concentrations of these molecules become high enough to induce fission. In addition, under physiological conditions LPA and PA have strongly positive and negative spontaneous curvatures, respectively^32^; thus the conversion from LPA to PA may generate negatively curved PA microdomains within overall positively curved membrane areas, which might lead to membrane destabilization. And, finally, the formation of PA might lead to other potential fission-related lipid-based mechanisms, such as the enzymatic conversion of PA into diacylglycerol^57^,^64^,^65^.

The precise role of the lipid and protein players considered in this study must now be defined. Most of the key components involved in BARS-dependent fission are available in pure form, and the possibility to reconstitute this fission pathway in artificial membranes using known components appears to be within reach.

## Methods

### Plasmids and chemicals and recombinant proteins

Human LPAAT cDNAs were from ImaGenes GmbH (for subcloning and mutations, see Supplementary Table 1); tdTomato-C1 cloning vector was from Addgene; BARS-pCDNA3, BARS–yellow fluorescent protein (BARS–YFP), BARS^S147A^–YFP, BARS^S147D^–YFP and BARS^D355A^–YFP were prepared as previously described^14^,^15^,^17^; E^0^GFP was provided by L. Marchetti (Scuola Normale Superiore, Pisa, Italy); LDLR^Y18A^–GFP was provided by R. Polishchuk (TIGEM, Naples, Italy); the HeLa hGH–GFP–FM-expressing cell line was provided by A. Peden (the University of Sheffield, Sheffield, UK). CI-976 was from Tocris Bioscience, tannic acid and BFA from Fluka, protease inhibitors as Complete Mini EDTA-free tablets from Roche, cycloheximide, Protein A Sepharose, anti-FLAG M2 affinity gel antibody beads and 3 × FLAG peptide from Sigma-Aldrich, AP21998 (D/D solubilizer) from Clontech, oleoyl-LPA from Avanti Polar Lipids, [^14^C]oleoyl-coenzyme A (specific activity, 60 mCi mmol^−1^) and dioleoyl [^14^C]-PA (specific activity, 140 mCi mmol^−1^) from PerkinElmer, and TRICH-labelled dextran, FITC-labelled dextran and MitoTracker Orange CM-H2TMRos from Molecular Probes. NAD^+^, BAC and HeLa (CD38+) cells were described previously^28^. Ni-NTA agarose and glutathione Sepharose beads were from Amersham, and Protein A Gold was from Cell Microscopy Center (the University Medical Center Utrecht, the Netherlands). Recombinant purified GST and GST-BARS proteins were produced from *E. coli* XL1Blue transformed cells with pGEX4T1 or pGEX4T1-BARS cells, respectively, grown at *A*_600_=0.4 before induction with 0.1 mM isopropyl-β-_D_-1-thiogalactopyranoside for 2 h at 37 °C (described in detail in ref. 55). Full-length and partial GST-LPAATδ (aa 152–251) purified by *in vitro* wheat germ expression system were from Antibodies-Online.com (catalogue number: ABIN1344584). His-*Ec*LPAAT (His-plsC) purifed *in vitro* wheat germ expression system was from Cusabio (catalogue number: CSB-EP34083ENV). *LPAATδ* siRNAs (M-008620-00), *LPAATγ* siRNAs (M-009283-01) and *BARS* siRNAs (M-008609-02) were from Dharmacon.

### Antibodies

All of the individually sourced antibodies for western blotting (WB), immunofluorescence and the LPAAT assay were obtained and used as detailed in ref. 15, unless otherwise stated. The rabbit polyclonal anti-LPAATδ (ab188002, 1:1,000) and anti-giantin (ab24586, 1:5,000) antibodies (for WB use) were from Abcam; the mouse polyclonal anti-LPAATδ antibody (for microinjection and LPAAT assay use) was from Abnova (H00056895-B01P); the rabbit polyclonal anti-LPAATδ antibody (1:50 for IF use) was from Sigma-Aldrich (SAB4502436), the anti-GM130 monoclonal antibody (1:200 for IF use) was from BD Transduction Laboratories (610823) and the rabbit polyclonal anti-Calnexin antibody (1:500 for WB use) was from Santa Cruz (AF18 sc23954). The anti-LPAATδ polyclonal antibodies from Abcam and Abnova recognize specifically LPAATδ and not other LPAATs by WB analysis (our unpublished data). Commercially available antibodies used: anti-TGN46 1:400 for IF use (Biorad, AHP500GT); anti-Giantin 1:500 for IF use (Alexis Corporation, 804-600-C100); anti-Flag 1:5,000 for WB and 1:500 for IF use (Sigma, F1804); anti-GAPDH 1:50,000 for WB use (ABD Serotec, MCA4740); anti-penta-His 1:1,000 for WB use (Life Technologies, P21315); anti-LPAATγ 1:1,000 for WB use (Abcam, ab93181). Source images from relevant WB are available in the Supplementary Figs 11–13.

### Immunoprecipitations and pull-down assays

HeLa cells in 10-cm Petri dishes were transiently transfected with 7 μg of each DNA (BARS-pCDNA3 and LPAATs–Flag) using 42 μl TransIT-LT1 per dish. Twenty-four hours after transfection, the cells were washed three times with phosphate-buffered saline (PBS) and lysed using 1 ml lysis buffer/dish (25 mM Tris, pH 7.4, 150 mM NaCl, 5 mM EDTA, 5 mM MgCl_2_, 10 mM NaF, 40 mM β-glycerophosphate, 1 mM Na_3_VO_4_, 1 mM dithiothreitol) supplemented with 1% Triton X-100 and protease inhibitor mixture (30 min, 4 °C, shaking). The lysates were centrifuged (13,000*g*, 10 min, 4 °C), with the supernatants assayed for protein concentration (Bradford assay) and used fresh.

For BARS immunoprecipitation, 500 μg lysate protein from these HeLa cells was brought to 0.2% (v/v) Triton X-100 (final concentration), and incubated with 3 μg anti-BARS polyclonal antibody (overnight, 4 °C, shaking)^55^. Then 50 μl protein A Sepharose beads were added for a further 1 h of incubation (4 °C, shaking). For LPAAT immunoprecipitation, 1.2 mg lysate protein from the HeLa cells was brought to 0.2% (v/v) Triton X-100 (final concentration), and incubated with 40 μl anti-FLAG M2 affinity-gel-purified antibody (2 h, 4 °C, shaking). For BARS immunoprecipitation in the presence of *Ec*LPAAT, 0.8 mg lysate protein from the COS7 cells was brought to 0.2% (v/v) Triton X-100 (final concentration) and incubated with 160 μg purified *Ec*LPAAT (2 h, 4 °C, shaking) and then incubated with 3 μg anti-BARS polyclonal antibody (overnight, 4 °C, shaking). The immune complexes were collected by centrifugation (500*g*, 5 min, 4 °C). After three washes with lysis buffer with 0.2% Triton X-100, and twice with lysis buffer without Triton X-100, the bound protein was eluted from the protein A Sepharose beads or from anti-FLAG M2 affinity-gel-purified antibody by boiling (10 min) in 100 μl Laemmli buffer. Thirty micrograms of input and 70% of the eluted proteins were separated by 10% SDS–PAGE, and subjected to WB via transfer to nitrocellulose membranes (Millipore).

For histidine pull-down from LPAATδ–Flag expressing cell lysate, His-BARS (20 μg) was incubated for 3 h at 37 °C with buffer alone (20 mM Tris, pH 7.4, 10 mM sucrose) or with 120 μM HPLC-purified BAC, to allow binding of BAC to His-BARS. The reaction mixture was stopped on ice, and 1 mg lysate protein from LPAATδ–Flag expressing cells was incubated with each sample (2 h, 4 °C, shaking). Then, 30 μl Ni-NTA agarose beads were added, and the samples were incubated (1 h, 4 °C, shaking). The beads were then washed three times with lysis buffer at pH 8.0 supplemented with 0.2% (v/v) Triton X-100 and 20 mM imidazole, by centrifugation (700*g*, 5 min), and then twice with lysis buffer at pH 8.0 without Triton X-100 but supplemented with 20 mM imidazole. The bound protein was eluted from the Ni-NTA agarose beads by boiling (10 min) in 100 μl Laemmli buffer. Twenty micrograms of input and 50% of the eluted proteins were separated by 10% SDS–PAGE, and subjected to WB via transfer to nitrocellulose membranes.

For GST pull-down with LPAATδ, 3 μg of His-BARS were incubated with 1.5 μg GST as control or with 4 μg GST-LPAATδ full length or with 3 μg GST-LPAATδ (aa 152–251) in GST incubation buffer (50 mM Tris, pH 8.0, 100 mM KCl, 0.2% Triton X-100, protease inhibitors; 2 h, 4 °C, shaking). Then, 30 μl glutathione sepharose beads were added for a further incubation (1 h, 4 °C, shaking). The beads were then washed five times with GST incubation buffer, by centrifugation (500*g*, 5 min). The bound protein was eluted from the glutathione sepharose beads with GST elution buffer (100 mM Tris, pH 8.0, 20 mM glutathione, 5 mM dithiothreitol). The 10% and 50% of the unbound and eluted proteins, respectively, were separated by 10% SDS–PAGE, and subjected to WB via transfer to nitrocellulose membranes.

For GST pull-down with *Ec*LPAAT, 3 μg of *Ec*LPAAT were incubated with 3 μg GST as control or with 5 μg GST-BARS in GST incubation buffer (20 mM Tris, pH 8.0, 1 mM EDTA, 0.2% Triton X-100, 100 mM KCl) (overnight, 4 °C, shaking). Then, 30 μl glutathione sepharose beads were added for a further incubation (1 h, 4 °C, shaking). The beads were then washed five times with GST incubation buffer, by centrifugation (500*g*, 5 min). The bound protein was eluted from the glutathione sepharose beads with GST elution buffer (100 mM Tris, pH 8.0, 20 mM glutathione, 5 mM dithiothreitol). Fifty per cent of the eluted proteins were separated by 10% SDS–PAGE, and subjected to WB via transfer to nitrocellulose membranes. For the GST pull-down with BAC-treated BARS, 5 μg GST-BARS was initially incubated with buffer alone (20 mM Tris, pH 7.4, 10 mM sucrose) or with 120 μM HPLC-purified BAC^28^ (3 h, 37 °C), to allow binding of BAC to GST-BARS in the GST incubation buffer. For the GST pull-down with NAD-treated BARS, 5 μg GST-BARS was initially incubated with 100 μM NAD^+^ in GST incubation buffer (1 h, room temperature). Full-scan images of all WB data are reported in Supplementary Figs 11–13.

### Transport protocols and light and wide-field microscopy

For the TGN-exit assay of VSVG, the cells were transfected with VSVG–GFP cDNA or infected with VSV, then incubated for 2 h at 40 °C, followed by 2 h at 20 °C (with 100 μg ml^−1^ cycloheximide) to accumulate VSVG in the Golgi complex. For the TGN-exit assay of p75, the cells were transfected with p75–GFP cDNA, incubated overnight in complete medium and for 2 h at 20 °C (with 100 μg ml^−1^ cycloheximide). To quantify VSVG-containing carriers, the TGN-exit assay was carried out in the presence of 0.5% tannic acid that inhibits fusion of the mature carriers to the plasma membrane and therefore leads to the accumulation of these carriers near the cell surface. The cells were then fixed and labelled with the P5D4 anti-VSVG antibody and the anti-TGN46 antibody. The fluorescent spots (0.5–2.0 μm) stained by both antibodies (at least 80% of VSVG-containing carriers) were then counted. The p75–GFP-containing carriers were counted as above, although without the co-staining for TGN46. All values are means±s.d. from three independent experiments. For the COPI transport assay, the cells were transfected with VSVG-ts045-KDELR-myc and then equilibrated at the permissive temperature of 32 °C for 3 h with the subsequent redistribution of the misfolded VSVG-KDELR chimera into the ER by shifting to the non-permissive temperature of 40 °C. The cells were immunostained with anti-VSVG antibody and the percentage of the cells with the ER staining was quantifed^19^. For the transport of the endocytosis-defective LDL–GFP receptor (LDLR^Y18A^), the cells were transfected with LDLR^Y18A^–GFP constructs and then equilibrated 2 h at 20 °C to accumulate the protein in the TGN with the subsequent shifting to 37 °C to allow for synchronized release of the proteins from the TGN^66^. For the hGH–GFP–FM transport, the HeLa cells stably transfected with hGH-FM–GFP were treated with the DD-solubilizer at 37 °C to induce the release of the protein from the ER to the PM^48^. The CI-976 treatment was performed during the VSVG TGN-exit assay, at 50 μM for 10 min, before the 32 °C temperature-release block and during the 32 °C temperature-release block. The anti-LPAATδ antibody (1 μg ml^−1^) was microinjected 1 h after the beginning of the 20 °C incubation in the VSVG transport assay, and after 1 h of recovery the cells were then processed for wide-field microscopy. Wide-field microscopy was performed as described previously^15^, with some modifications. COS7 cells were transfected with siRNAs for LPAATδ (Smart Pool, Lipofectamine 2000), and after 48 h the cells were transfected with VSVG–cyan fluorescent protein (VSVG–CFP; overnight, 40 °C) and then incubated with 100 μg ml^−1^ cycloheximide (3 h, 20 °C). The cells were then shifted to 32 °C (with continued cycloheximide), and followed by fast video microscopy (see ref 15). For CI-976 treatment, COS7 cells were treated with 50 μM CI-976 for 10 min before the shift to 32 °C.

### FLIM measurements

The DNA coding for E^0^GFP–BARS and LPAATδ-tdTomato were generated using the Restriction Free (RF) cloning procedure^67^ using the following two pairs of primers: 5′-cacgctggccggagagccttatgtcaggcgtccgacct-3′ and 5′-tttaaacgggccctctagagcactacaactggtcagtcgta-3′; and 5′-ctaccggactcagatctcgagatggacctcgcgggac-3′ and 5′-cccttgctcaccatggtggcctgattcagtttctggttg-3′, respectively. *BARS* and *LPAATδ* genes were amplified as follows: a single denaturation step (95 °C, 5 min), followed by 35 cycles PCR (95 °C, 1 min; 50 °C, 30 s; 72 °C, 2 min) and a final elongation step of 5 min (72 °C), in a final volume of 50 μl (including 100 ng of template DNA, 0.4 μM of each primer, 200 μM of each dNTPs, 1 × Pfu buffer and 2 U Pfu Turbo Cx hotstart DNA polymerase, Agilent). Then the amplified genes were purified using the Agilent Strataprep PCR purification kit and used as mega-primer in the second PCR for gene cloning. LPAATδ was cloned into tdTomato-N1 vector, fused to the N-terminal of tdTomato sequence, and BARS was cloned into pcDNA3.1+ vector carrying the E^0^GFP sequence^68^ fused to the C-terminal of E^0^GFP sequence. The RF reactions were carried out as follows: a single denaturation step (95 °C, 5 min) was performed followed by 20 cycles PCR (95 °C, 1 min; 50 °C, 30 s; 72 °C, 7 min) and a final elongation step of 10 min at 72 °C. RF reactions were performed in a final volume of 50 μl (including 50 ng of target DNA, 300 ng of PCR mega-primer products, 200 μM of each dNTPs, 1 × Pfu buffer and 5 U Pfu Turbo Cx hotstart DNA polymerase). Thirty microlitre of each PCR mix were digested using 20 U *Dpn*I (37 °C, 2 h), and 10 μl of each aliquot were used to transform *E. coli* Top10 strain (Invitrogen). Colony PCR screening were used to search positive clones by using specific primers for BARS and LPAATδ. The cloned fragments were then completely sequenced and verified.

COS7 cells were transiently transfected with 0.4 μg E^0^GFP–BARS and 0.4 μg LPAATδ-tdTomato using *Trans*IT-LT1 Transfection Reagent, according to manufacturer's instructions. Sixteen hours after transfection, the cells were subjected to the VSVG TGN-exit assay. After 10 min of the 32 °C temperature-release block, the cells were fixed with 4% (w/v) PFA in 20 mM Hepes pH 7.4, for 10 min at room temperature. The samples were then treated with 100 mM glycine in 20 mM Hepes for 30 min, washed three times with 20 mM Hepes and mounted in glycerol/20 mM Hepes (1:1).

The lifetime values were extracted from the fitting of decay curves obtained from the donor emission (E^0^GFP–BARS) on the Golgi area, and global and pixel kinetic data analyses are computed by a commercially available software package SymPho Time, Version 5.3.2.2 (PicoQuant GmbH, Berlin, Germany). Time-resolved images were acquired by the confocal microscope Leica SP5 II equipped with the White Light Laser (both Leica Microsystems GmbH, Mannheim, Germany) and the TCSPC measurements were performed with the SMD Upgrade Kit (PicoQuant GmbH). The excitation light was pulsed at 80 MHz and the donor fluorophore was excited at 475 nm and acquired from 495 to 530 nm. The measurements were carried out in fixed cells, for ease and speed of acquisition of a statistically reliable number of samples. The donor lifetime values were extracted from the fitting of decay curves obtained from the donor emission (E^0^GFP–BARS) in the Golgi area, and both global and pixel kinetic data analyses were computed by the commercially available software package SymPho Time, Version 5.3.2.2. Changes in donor lifetime due to FRET were assessed by comparing the donor lifetime in cells expressing only the donor with the lifetime in cells co-transfected with donor and acceptor and fixed either under steady-state condition or during a traffic pulse.

### Cryo-immunogold electron microscopy

HeLa cells were transiently transfected with 8 μg plasmid DNA encoding Flag-LPAATδ for 24 h (using TransIT-LT1) and then fixed with 2% formaldehyde and 0.2% glutaraldehyde in 100 mM Phosphate buffer pH 7.4. The cells were pelleted by centrifugation, embedded in 12% gelatin, cooled on ice and cut into 1-mm^3^ cubes at 4 °C. The cubes were immersed in 2.3 M sucrose at 4 °C overnight, frozen in liquid nitrogen and cut using Leica EM FC7 ultramicrotome^69^. Thin sections (50 nm) were picked up in a mix of 2% methylcellulose and 2.3 M sucrose (1:1) and incubated with the rabbit anti-LPAATδ (1:10) and the mouse anti-Golgin-97 (Life Technologies, catalogue number: A21270, 1:50) antibodies. Each of these incubations was followed by incubations with Protein A gold; 10 nm Gold particles for anti-LPAATδ; and 15 nm Gold particles for anti-Golgin-97. After labelling, the sections were treated with 1% glutaraldehyde and embedded in methylcellulose uranyl acetate. Electron microscopy images were acquired using FEI Tecnai-12 electron microscope.

### Transfections with siRNAs

COS7 and HeLa cells were transfected with a non-targeting siRNA or with 150 nm of a Smart Pool of *LPAATδ/*M-009283 or *LPAATγ*/M-008620 siRNAs, for 72 h (except for *BARS* and *14-3-3s* siRNAs, where 100 nm of a Smart Pool was used for 48 h, as previously described in ref. 15) using Lipofectamine 2000, according to manufacturer's instructions. The efficiency of interference was assessed by WB. The treatment with Smart Pool siRNAs for LPAATγ (M-008620) and for LPAATδ (M-009283) specifically reduces the endogenous protein levels of LPAATγ and LPAATδ (by WB), respectively, without affecting the levels of other tested LPAATs (our unpublished data). Alternatively, COS7 cells were transfected with the siRNAs (as above) in combination for the last 16 h with VSVG–CFP, VSVG–GFP, VSVG-ts045-KDELR-myc, LDLR^Y18A^–GFP or p75–GFP, and then subjected to the specified Golgi-transport assay^14^,^15^,^66^. For the rescue experiments, COS7 cells were transfected with siRNAs for LPAATδ/D-009283-03 (5′-GCACACGGUUCACGGAGAA-3′, Dharmacon) for 48 h, and transfected for a further 24 h (using TransIT-LT1) with Flag-LPAATδ^wt^ or Flag-LPAATδ^H96V^ (both encode an siRNA-resistant silent mutation), followed by infection with VSV for the TGN-exit assay.

### *In vitro* acyltransferase assay

HeLa cells (1 × 10^6^) in 10-cm Petri dishes were transiently transfected with 8 μg plasmid DNA encoding Flag-LPAATδ^wt^ or Flag-LPAATδ^H96V^ for 48 h (using TransIT-LT1). Alternatively, the HeLa cells were transfected with siRNAs (as above) in combination with Flag-LPAATδ^wt^ for 48 h (using Lipofectamine 2000). The cells were washed three times with PBS, harvested as 250 μl per dish in homogenization buffer (100 mM Tris, pH 7.4, 5 mM NaCl, 3 mM MgCl_2_) supplemented with the protease inhibitor mixture, and homogenized (6 pulses, 30% amplitude; Branson Digital Sonifier). The lysate was centrifuged at 600*g* for 10 min at 4 °C, and the post-nuclear supernatant fraction was used in the acyltransferase assay.

Golgi membranes from HeLa cells treated as described above were obtained as described previously^70^, with some modifications. HeLa cells were washed with PBS, harvested by trypsinization and pelleted (400*g*, 5 min, 4 °C). The cell pellets were resuspended in 1:4 vol. homogenization buffer (250 mM sucrose, 100 mM Tris, pH 7.4) supplemented with the protease inhibitor mixture. The cells were then passed six times each direction (12 passes total) through the Balch homogenizer with a 7.988 mm diameter tungsten-carbide ball bearing (clearance, 12 μm) using constant manual pressure. Following homogenization, the samples (≈ 850 μl in 0.25 M sucrose, 10 mM Tris-HCl, pH 7.4) were adjusted to 1.4 M sucrose by addition of ice-cold 2.3 M sucrose containing 10 mM Tris-HCl (pH 7.4). Then, 1 mM Na_2_EDTA was added from a 100 mM stock solution, and the samples were vortexed vigorously to ensure uniform mixing. The suspension (≈ 2.8 ml) was layered onto sucrose gradients (700 μl 2.0 M and 1.4 ml 1.6 M sucrose, from the bottom of the tubes for a swing-out rotor) and covered with 4.2 ml 1.2 M and 2.8 ml 0.8 M sucrose, from the suspension fraction. After centrifugation (90,000*g*, 2.5 h, 4 °C), the Golgi-enriched fraction was collected at the 0.8/1.2 M sucrose interface, aliquoted, frozen in liquid nitrogen and stored at −80 °C.

Two micrograms of the post-nuclear supernatant or of the purified Golgi membranes fractions were incubated with the LPAAT reaction buffer (75 mM Tris, pH 7.4, 4 mM MgCl_2_, 1 mM dithiothreitol, 4 mM NaF, 1 mg ml^−1^ bovine serum albumin fatty acid free, 50 μM oleoyl-LPA, 20 μM [^14^C]oleoyl-CoA) in a final volume of 100 μl, for 20 min at 25 °C. The total lipids were extracted by adding 450 μl cold CHCl_3_/CH_3_OH (2:1, v/v). After 30 min on ice, the samples were centrifuged (10,000*g*, 5 min). The lower, organic, phase was dried under a stream of N_2_, resuspended in 50 μl CHCl_3_, and loaded onto an oxalate-pretreated TLC plate^55^. The lipids were separated by running the TLC plates with CHCl_3_/CH_3_OH/33% NH_4_OH/H_2_O (54:42:2.9:9.1; v/v/v/v). The radiolabelled spots were quantified by gas ionization counting (Beta-Imager Systems, Biospace Laboratories). Dioleoyl [^14^C]-PA was used as a standard.

For CI-976 treatment, the post-nuclear fraction from HeLa cells was incubated with 50 μM CI-976 for 30 min at 25 °C, followed by the addition of the LPAAT reaction buffer (as above). For anti-LPAATδ or anti-BARS antibody treatment, the post-nuclear or Golgi membrane fraction from HeLa cells was incubated with 100 ng anti-LPAATδ or anti-BARS affinity-purified polyclonal antibody for 30 min at 25 °C, followed by addition of the LPAAT reaction buffer (as above). For immunopurified BARS treatment, the post-nuclear fraction from HeLa cells was incubated with 500 ng immunoprecipitated BARS purified from rat-brain cytosol with anti-BARS-IgG crosslinked matrice, as described in ref. 15 for 30 min at 25 °C, followed by addition of the LPAAT reaction buffer (as above).

In these experiments, LPAATδ-dependent activity (or LPAATδ activity) is defined as the activity value of LPAATδ-overexpressing extracts minus the activity value of LPAATδ depleted (or antibody-treated) extracts (see also Fig. 5a). In Figs 5, 6, 7 and Supplementary Fig. 10, the LPAATδ-independent activity (that is, derived from LPAATδ-depleted or antibody-treated extracts) is indicated with a dashed line. The LPAATδ-independent activity was reproducibly ≈50% of the total activity in LPAATδ-overexpressing extracts (as evaluated in >30 independent experiments) and ≈30% of the total activity in purified Golgi membranes from LPAATδ-overexpressing cells (as evaluated in >20 independent experiments).

### Size-exclusion chromatography

For the fast protein liquid chromatography, 600 μg purified recombinant GST or GST-BARS was applied to a Sephacryl S-200 High Resolution HiPrep 16/60 (Amersham Pharmacia) gel filtration column equilibrated with PBS buffer (4 °C; flow rate, 0.3 ml min^−1^), with 1 ml fractions collected using an AKTA Fast Protein Liquid Chromatography (FPLC) system. The eluted protein was detected by monitoring absorbance at 280 nm, and 10 μl of the collected fractions was separated on 10% SDS–PAGE gels, and analysed by silver staining. Eighty microlitres of each of these fractions was then subjected to the LPAAT assay.

### Ion-exchange chromatography

For the fast protein liquid chromatography, 600 μg purified recombinant GST-BARS was applied to a MonoQ column (HR5/5 Pharmacia LKB) equilibrated with buffer A (25 mM Tris, pH 8.00, 50 mM KCl; 4 °C; flow rate, 1 ml min^−1^), with 0.5 ml fractions collected using an AKTA FPLC system. After washing the MonoQ column with buffer A, the protein was eluted with a 20 ml gradient of 0.05–1 M KCl, and 10 μl of the collected fractions were separated on 10% SDS–PAGE gels and analysed by silver staining. Eighty microlitres of each of these fractions was then subjected to the LPAAT assay.

### Statistical analysis

Two-tailed Student's *t*-tests were applied to the data. Significance is indicated as *P<0.05, **P<0.01 and ***P<0.005.

### Data availability

The authors declare that the data supporting the findings of this study are available within the article and its Supplementary Information files or are available from the corresponding authors upon request.

## Additional information

**How to cite this article:** Pagliuso, A. *et al*. Golgi membrane fission requires the CtBP1-S/BARS-induced activation of lysophosphatidic acid acyltransferase δ. *Nat. Commun.* 7:12148 doi: 10.1038/ncomms12148 (2016).

## Supplementary Material

Supplementary Information Supplementary Figures 1-13 and Supplementary Table 1

Supplementary Movie 1 Export of VSVG-GFP from the Golgi complex in COS7 cells following non-targeting siRNAs. Following 48 h of non-targeting siRNAs treatment, COS7 cells were transfected for 24 h with VSVG-GFP, subjected to the TGN-exit assay, and observed at 32°C *in vivo* under confocal microscopy. Several VSVG-GFP- containing carriers can be seen to be formed from the Golgi complex and to move across the cell toward the plasma membrane.

Supplementary Movie 2 Export of VSVG-GFP from the Golgi complex in COS7 cells following LPAATd-targeting siRNAs. Following 48 h of LPAATd-targeting siRNAs treatment, COS7 cells were transfected for 24 h with VSVG-GFP, subjected to the TGN-exit assay, and observed at 32°C *in vivo* under confocal microscopy. Several post-Golgi carrier precursors can be seen to extend from the Golgi complex, but they do not undergo fission, resulting in long tubular carrier precursors. The arrowheads indicate some VSVG-GFP-containing carriers with aberrantly extended tubular shapes. The arrow indicates a carrier that after fission, moves towards, and fuses with, the plasma membrane.

Supplementary Movie 3 Post-Golgi carrier formation in VSVG-GFP expressing COS7 cells following anti-LPAATd antibody injection. VSVG-GFP-expressing COS7 cells were subjected to the TGN-exit assay, and after 1 h at 20°C the cells were microinjected with an anti-LPAATd antibody and incubated for a further 1 h at 20°C. The cells were then observed at 32°C *in vivo* under confocal microscopy. The microinjected cell shows long tubular carrier precursors (top right: indicated by the arrowhead; see also Supplementary Fig. 5).

Supplementary Movie 4 Post-Golgi carrier formation in VSVG-GFP-expressing COS7 cells following CI-976 treatment. VSVG-GFP-expressing COS7 cells were subjected to the TGN-exit assay and treated with the general LPAAT inhibitor CI-976 (50 μM, 15 min) before the 32°C temperature-block release. The cells were then observed *in vivo* under confocal microscopy. The CI-976 treatment dramatically reduces the fission of post-Golgi tubular carrier precursors, and increases the lengths of the fissioned postGolgi carriers.
