# Supplementary material for: Golgi membrane fission requires the CtBP1-S/BARS-induced activation of lysophosphatidic acid acyltransferase δ
Source: Nat Commun. 2016 Jul 12;7:12148. doi: 10.1038/ncomms12148 (PMC4945875; doi:10.1038/ncomms12148)
Supplement: Supplementary Information — Supplementary Figures 1-13 and Supplementary Table 1 [file ncomms12148-s1.pdf]

Supplementary Fig. 1

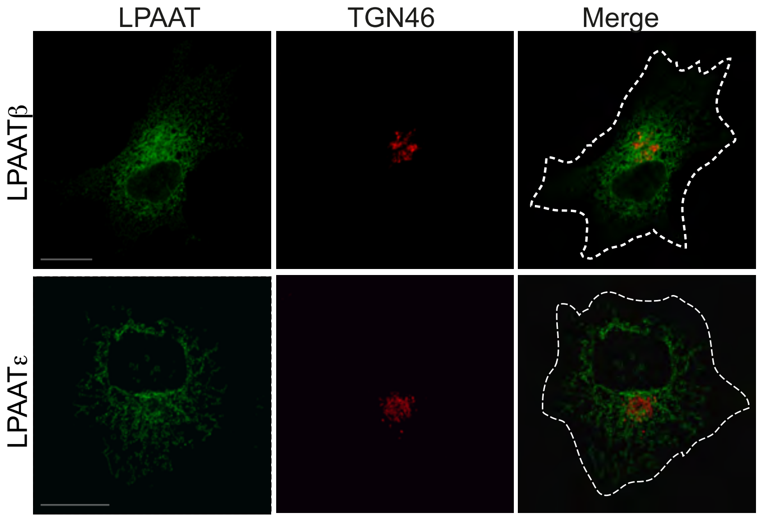

Supplementary Figure 1. Localization of LPAATβ and LPAATε. Representative confocal microscopy images of COS7 cells transfected with Flag-tagged LPAATβ and LPAATε (as indicated) for 24 h, and fixed and processed for immunofluorescence with a monoclonal anti-Flag antibody (green; LPAAT) and with a polyclonal anti-TGN46 antibody (red; TGN). Dotted lines indicate cell borders. Scale bars: 10 μm.

Supplementary Fig. 2

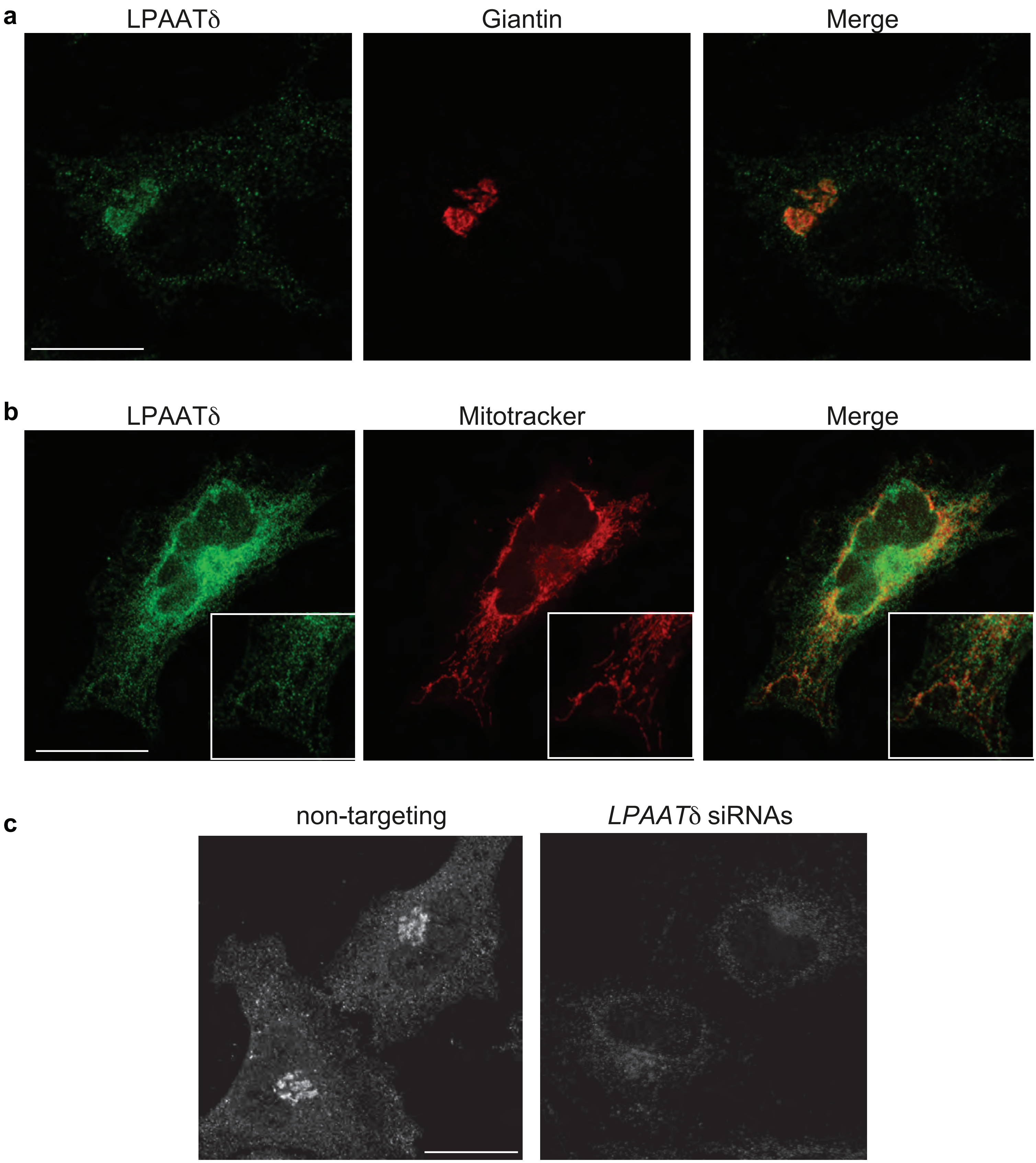

Supplementary Figure 2. Localization of endogenous LPAATδ. (a,b) Representative confocal microscopy images of HeLa cells fixed and stained with the anti-LPAATδ (SAB1101918, in green) at low antibody titer (15 ng μl^{-1}; **a**) or at higher antibody titer (80 ng μl^{-1}; **b**) and anti-giantin antibodies (in red), as indicated. Inset in **b**, bottom: magnification of mitochondria staining. Of note, similar results were obtained in COS7, NIH3T3 and HEK cells. **(c)** Representative confocal microscopy images of COS7 cells transfected with non-targeting or LPAATδ siRNAs, fixed and labelled with the anti-LPAATδ (at higher antibody titer, as above). Note that in LPAATδ-knockdown cells both the signals at the Golgi and at the mitochondria are almost completely abolished. Scale bar: 10 µm.

Supplementary Fig. 3

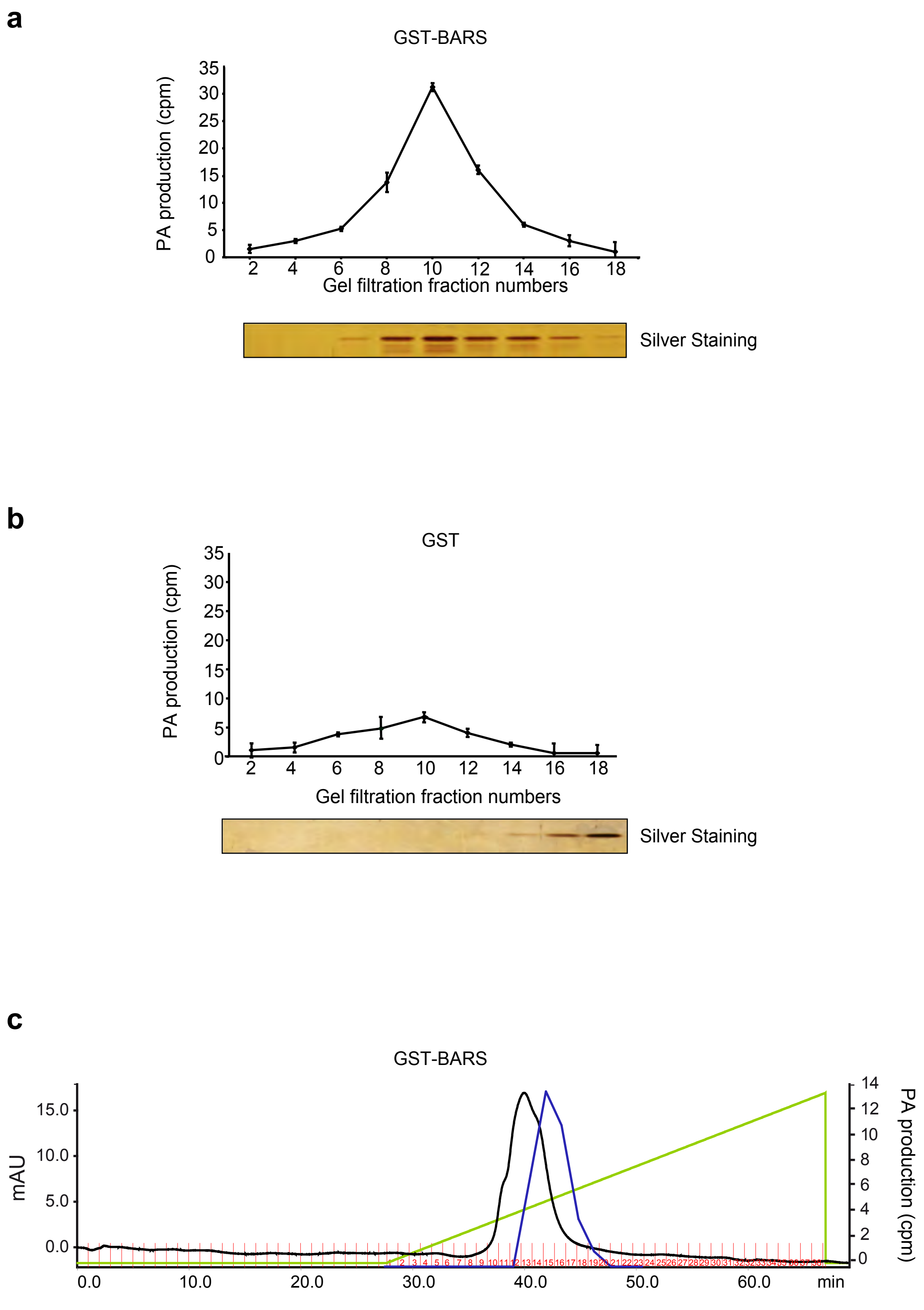

Supplementary Figure 3. Purified recombinant BARS is associated with an LPAAT activity. (a,b) Quantification of phosphatidic acid (PA) production in the LPAAT assay for purified recombinant GST-BARS in **a**, and GST in **b** fractions (as indicated) with PBS elution from the size-exclusion chromatography column (Sephacryl S-200 column), at 0.3 ml min^{-1} at 4°C. Aliquots of each fraction were subjected to the LPAAT assay and the production of PA was analyzed and quantified. Bottom: Silver staining analysis of the protein elution pattern of GST-BARS in **a** and GST in **b** in the fractions after size-exclusion chromatography. **(c)** Representative monoQ ion-exchange chromatography profiles of purified recombinant GST-BARS, monitoring absorbance at 280 nm (mAU; black line), as eluted with an NaCl gradient (green line). Aliquots of each fraction (as indicated in red) were subjected to the LPAAT assay, and the PA production is indicated by the blue line.

Supplementary Fig. 4

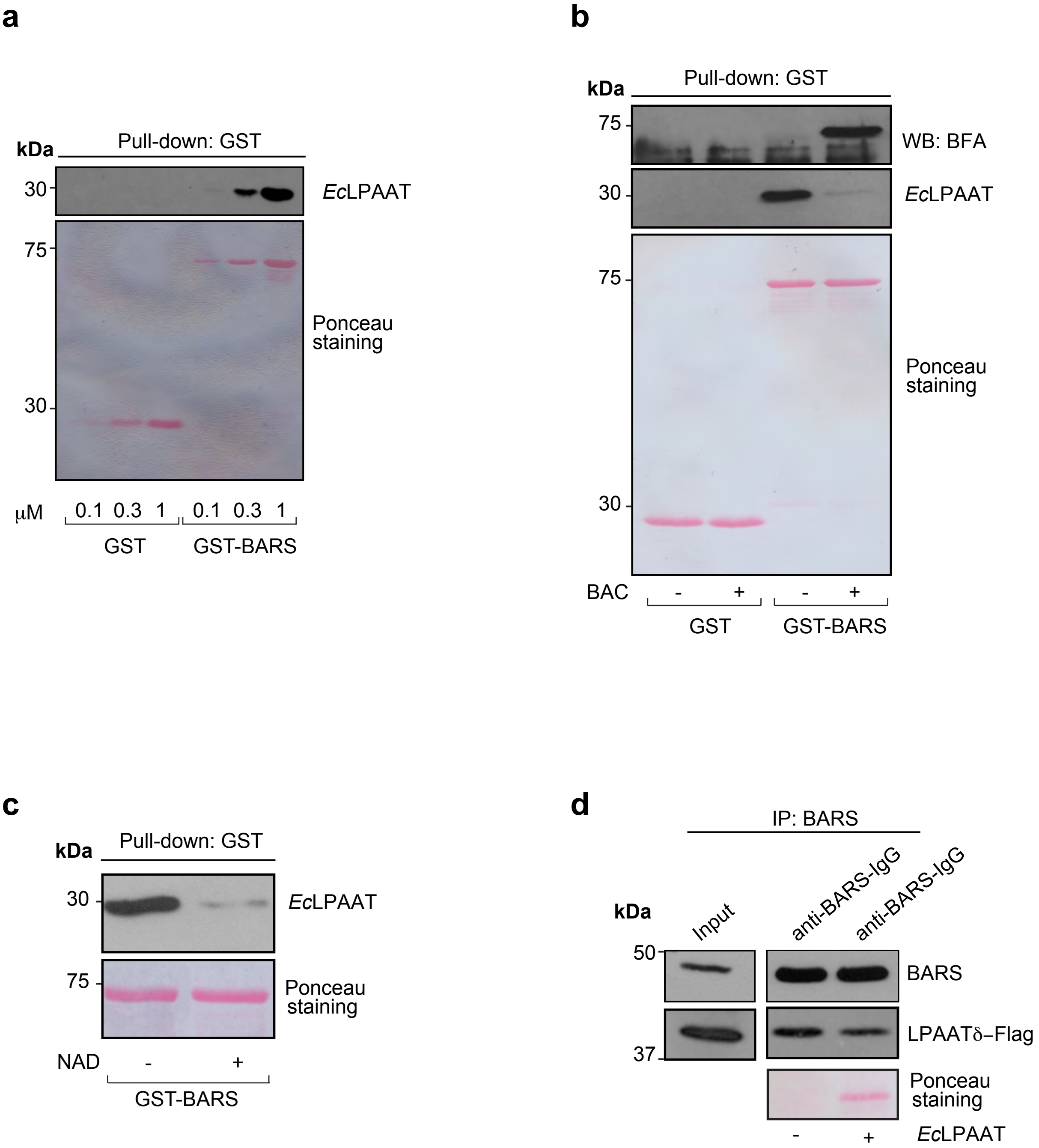

Supplementary Figure 4. BARS binds Escherichia coli LPAAT in a conformation-dependent manner. Representative GST pull-down assays for GST and GST-BARS beads (as indicated). **(a)** Using recombinant His-tagged E. coli LPAAT (EcLPAAT). **(b)** Using buffer alone (-) or HPLC-purified BAC (BAC+), and then incubated with recombinant EcLPAAT. **(c)** Using buffer alone (-) or 100 μM NAD+ (NAD+), and then incubated with recombinant EcLPAAT. The eluted proteins were analyzed by Western blotting (top) using an anti-histidine antibody to monitor EcLPAAT in a-c while the BARS-bound BAC was detected using an antibody developed against the BFA moiety of BAC that recognizes the BAC-BARS conjugate (WB:BFA)[28] in **b**. The pulled-down proteins were revealed by Ponceau-S staining (bottom). **(d)** Representative Western blotting with anti-BARS and anti-Flag antibodies for BARS immunoprecipitation (IP:BARS) of lysate from COS7 cells transfected with LPAATδ-Flag in the absence (-) or presence (EcLPAAT+) of recombinant purifed EcLPAAT with anti-BARS IgG. Total lysate (input) and BARS-immunoprecipitated protein are shown. Molecular weight standards (kDa) are indicated on the left of each panel.

Supplementary Fig. 5

a

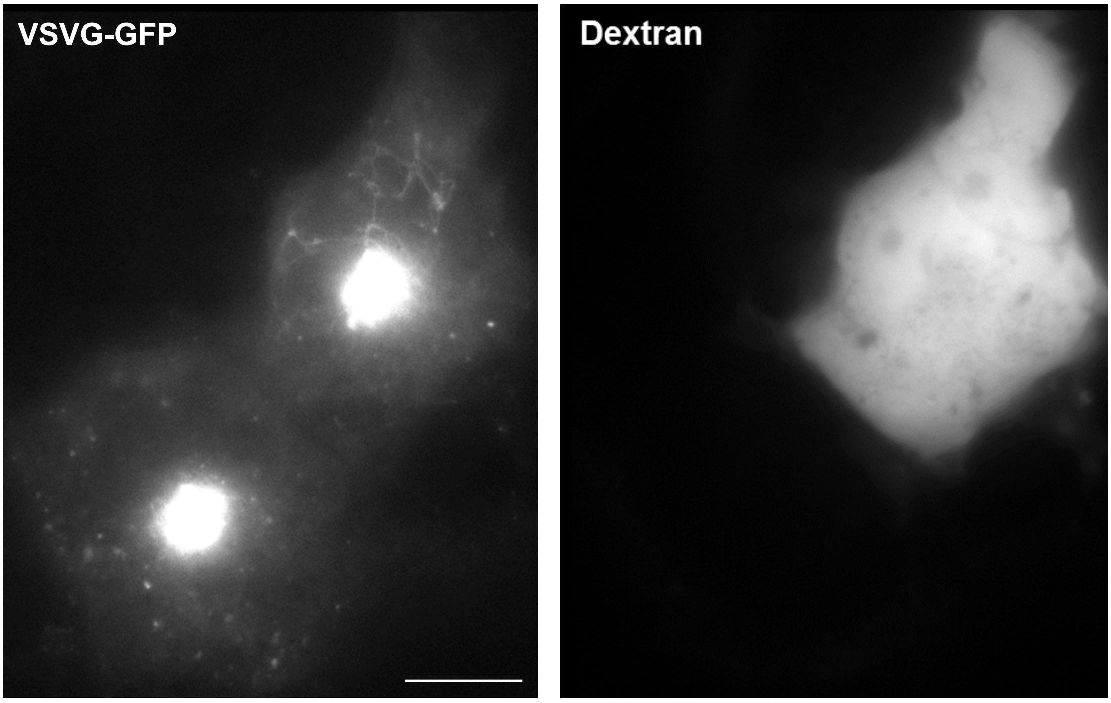

b

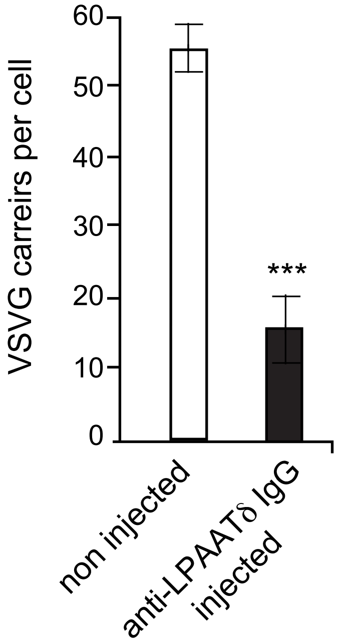

Supplementary Figure 5. Post-Golgi carrier formation in VSVG-GFP expressing COS7 cells following anti-LPAATδ antibody injection. (a) Snapshot of Supplementary Video 3 for post-Golgi carrier formation in VSVG-GFP–expressing COS7 cells (VSVG-GFP; left) after microinjection with an anti-LPAATδ antibody mixed with TRICH-dextran (dextran; right). Scale bar: 10 µm. **(b)** Quantification of VSVG-containing post-Golgi carriers in non-injected or anti-LPAATδ antibody-injected cells treated as in **a**.

Supplementary Fig. 6

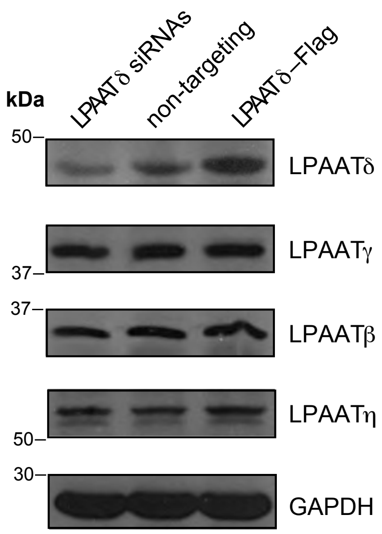

Supplementary Figure 6. Depletion and overexpression of LPAATδ in HeLa cells do not affect the endogenous levels of other LPAATs. Representative Western blotting with anti-LPAATδ, anti-LPAATγ, anti-LPAATβ, anti-LPAATη and anti-GAPDH antibodies (as indicated) of HeLa cells transfected with LPAATδ-siRNA or with non-targeting (non-targeting) for 48 h, or with LPAATδ-Flag for 24 h (as indicated). Molecular weight standards (kDa) are indicated on the left of each panel.

Supplementary Fig. 7

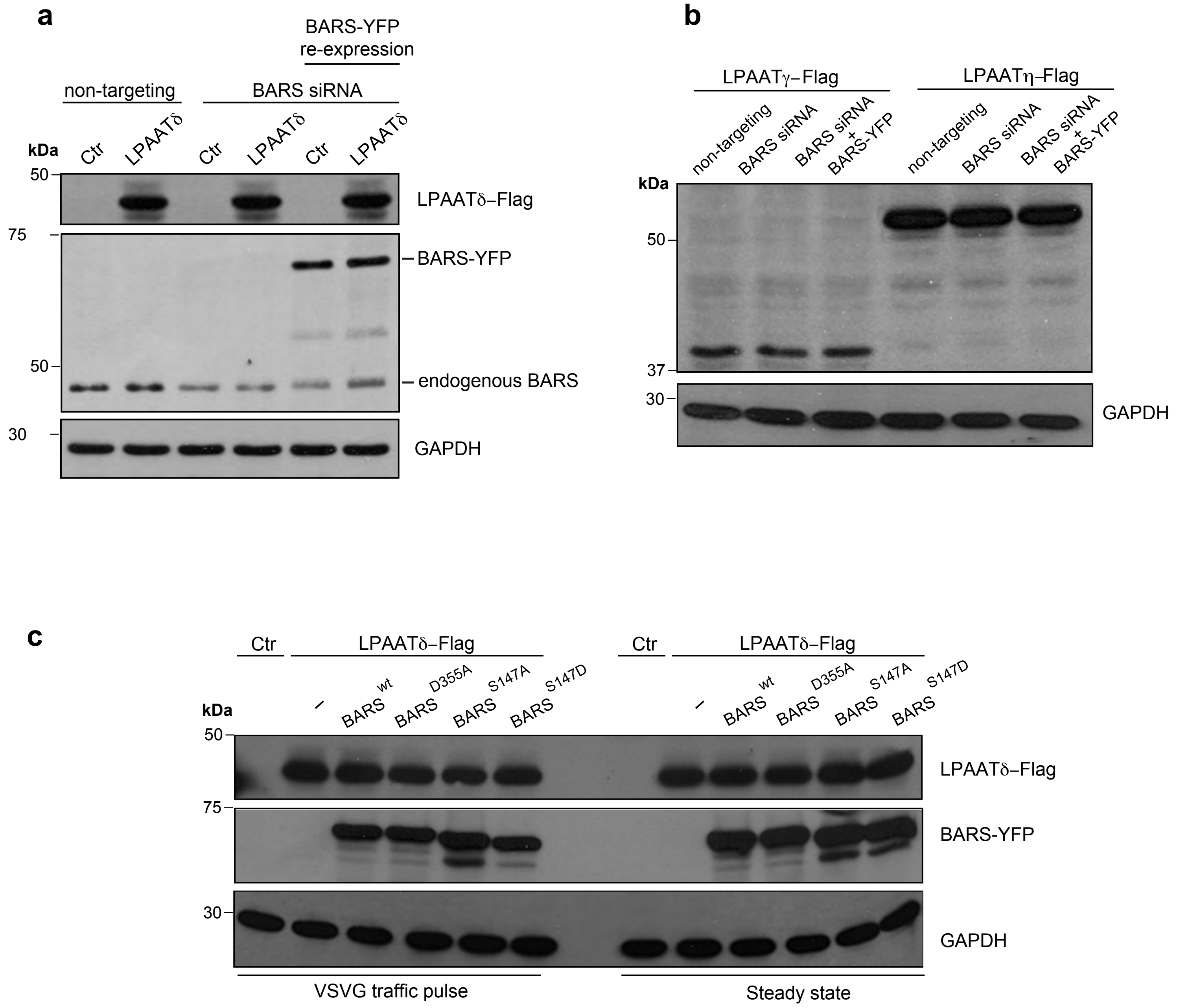

Supplementary Figure 7. Depletion and overexpression of BARS in LPAATδ-expressing HeLa cells. **(a)** Representative Western blotting with anti-Flag, anti-BARS, and anti-GAPDH antibodies (as indicated) of HeLa cells transfected with empty Flag-vector (Ctr) or LPAATδ-Flag (LPAATδ) and with non-targeting (non-targeting) or BARS siRNA (duplex #2) for 48 h, and with the last 12 h with a siRNA-resistant replacement BARS-YFP-encoding vector (BARS-YFP re-expression). **(b)** Representative Western blotting with anti-Flag and anti-GAPDH antibodies (as indicated) of HeLa cells transfected with LPAATγ-Flag or LPAATη-Flag and with non-targeting (non-targeting) or BARS siRNA (duplex #2) for 48 h, and with the last 12 h with a siRNA-resistant replacement BARS-YFP-encoding vector (BARS-YFP). **(c)** Representative Western blotting with anti-Flag, anti-BARS and anti-GAPDH antibodies (as indicated) of HeLa cells transfected with empty Flag-vector (Ctr) or LPAATδ-Flag (LPAATδ) for 48 h, and with the last 12 h with BARS^{wt}-YFP, BARSD^{355A}-YFP, BARS^{S147A}-YFP, or BARS^{S147D}-YFP, at steady state (right) or after a VSVG traffic pulse (left) (as indicated). Molecular weight standards (kDa) are indicated on the left of each panel.

Supplementary Fig. 8

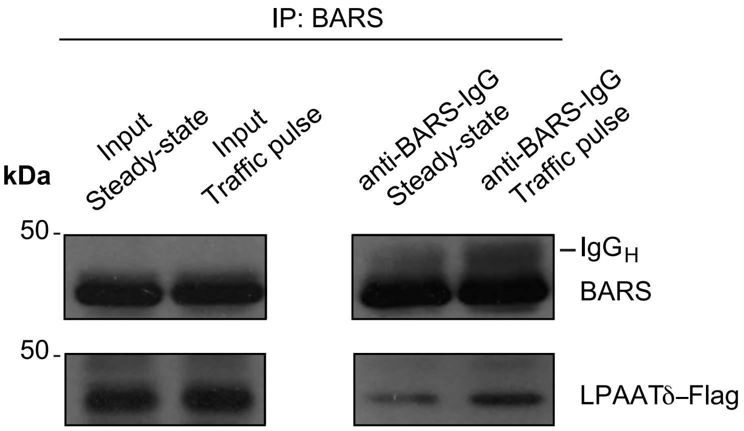

Supplementary Figure 8. The interaction between LPAATδ and BARS increases during the traffic pulse. BARS immunoprecipitation (IP:BARS) of lysate from HeLa cells co-expressing BARS and LPAATδ-Flag at the steady state or after 10 min of the 32°C temperature-release block in the VSVG-TGN exit assay (Traffic pulse), as indicated. Representative Western blotting (antibodies as indicated) of total lysate (Input) and immunoprecipitated proteins with anti-BARS-IgG (as indicated). IgGH, IgG heavy chain. Molecular weight standards (kDa) are indicated on the left of each panel.

Supplementary Fig. 9

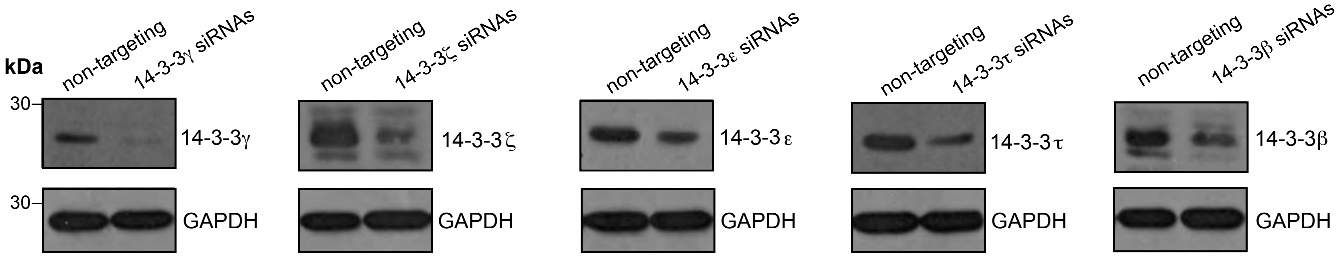

Supplementary Figure 9. Depletion of 14-3-3s in LPAATδ-expressing HeLa cells. Representative immunoblotting of HeLa cells transfected with non-targeting siRNA or 14-3-3γ, ζ, ε, τ and β siRNAs (as indicated) for 48 h with isoform-specific anti-14-3-3 antibodies and an anti-GAPDH antibody (for internal protein levels). Note that the HeLa cells transfected with both non-targeting siRNA and 14-3-3s siRNAs were co-transfected in combination with LPAATδ-Flag. Representative Western blotting with an anti-Flag antibody is shown in Figure 7b.

Supplementary Fig. 10

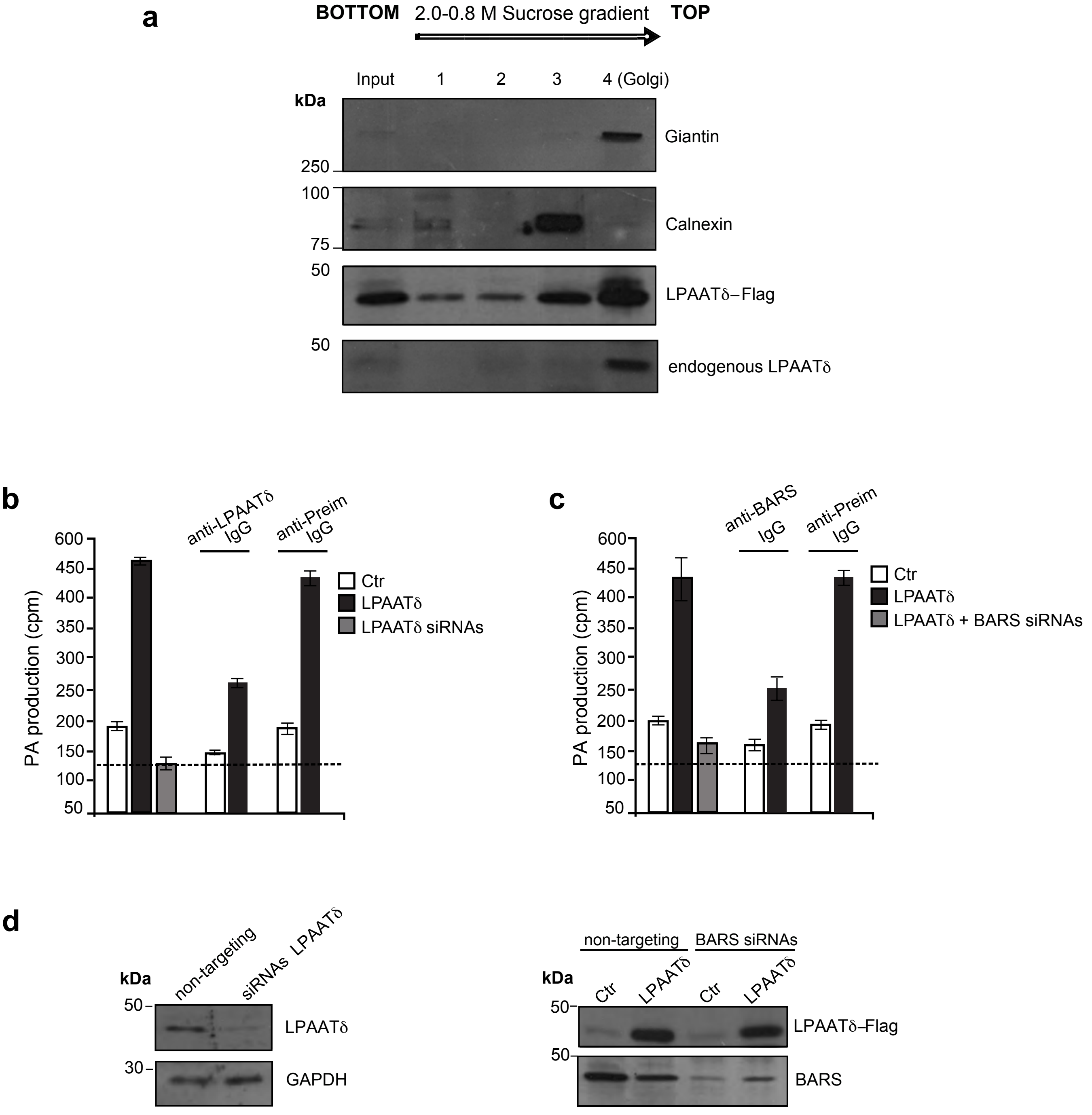

Supplementary Figure 10. Phosphatidic acid production from purified Golgi membranes. (a) Representative Western blotting with anti-giantin (Golgi marker), anti-calnexin (ER marker), anti-Flag, and anti-LPAATδ antibodies (as indicated), of post-nuclear supernatant from HeLa cells fractionated by equilibrium centrifugation on a sucrose gradient (0.8-2.0 M). Golgi membranes were enriched in gradient fraction #4 (1.2-0.8 M sucrose gradient). **(b)** Quantification of phosphatidic acid (PA) production in the LPAAT assay for Golgi membranes from HeLa cells as in **a**, transfected with empty Flag-vector (Ctr) or LPAATδ-Flag (LPAATδ) for 48 h or with LPAATδ siRNAs for 72 h. The anti-LPAATδ polyclonal antibody (anti-LPAATδ IgG) or anti-preimmune IgG (anti-Preim IgG, as control) were incubated with the indicated Golgi membrane fraction for 30 min at 25°C before LPAAT assay. **(c)** Quantification of phosphatidic acid (PA) production in the LPAAT assay for Golgi membranes from HeLa cells as in a, transfected with empty Flag-vector (Ctr) or LPAATδ-Flag (LPAATδ) and with BARS siRNAs for 48 h. The anti-BARS polyclonal antibody (anti-BARS IgG) or anti-preimmune IgG (anti-Preim IgG, as control) were incubated with the indicated Golgi membrane fraction for 30 min at 25°C before LPAAT assay. The dashed line indicates the level of endogenous LPAAT activity not associated with LPAATδ (see text for details). **(d)** Representative Western blotting with an anti-LPAATδ and anti-Flag antibody (as indicated), for the transfection and depletion efficiencies, of these proteins used for the LPAAT assays in **c** and **d**. Glyceraldehyde 3-phosphate dehydrogenase (GAPDH) is shown for the internal protein levels. Molecular weight standards (kDa) are indicated on the left of each panel. Data are means ± s.d. of three independent experiments.

Supplementary Fig.11

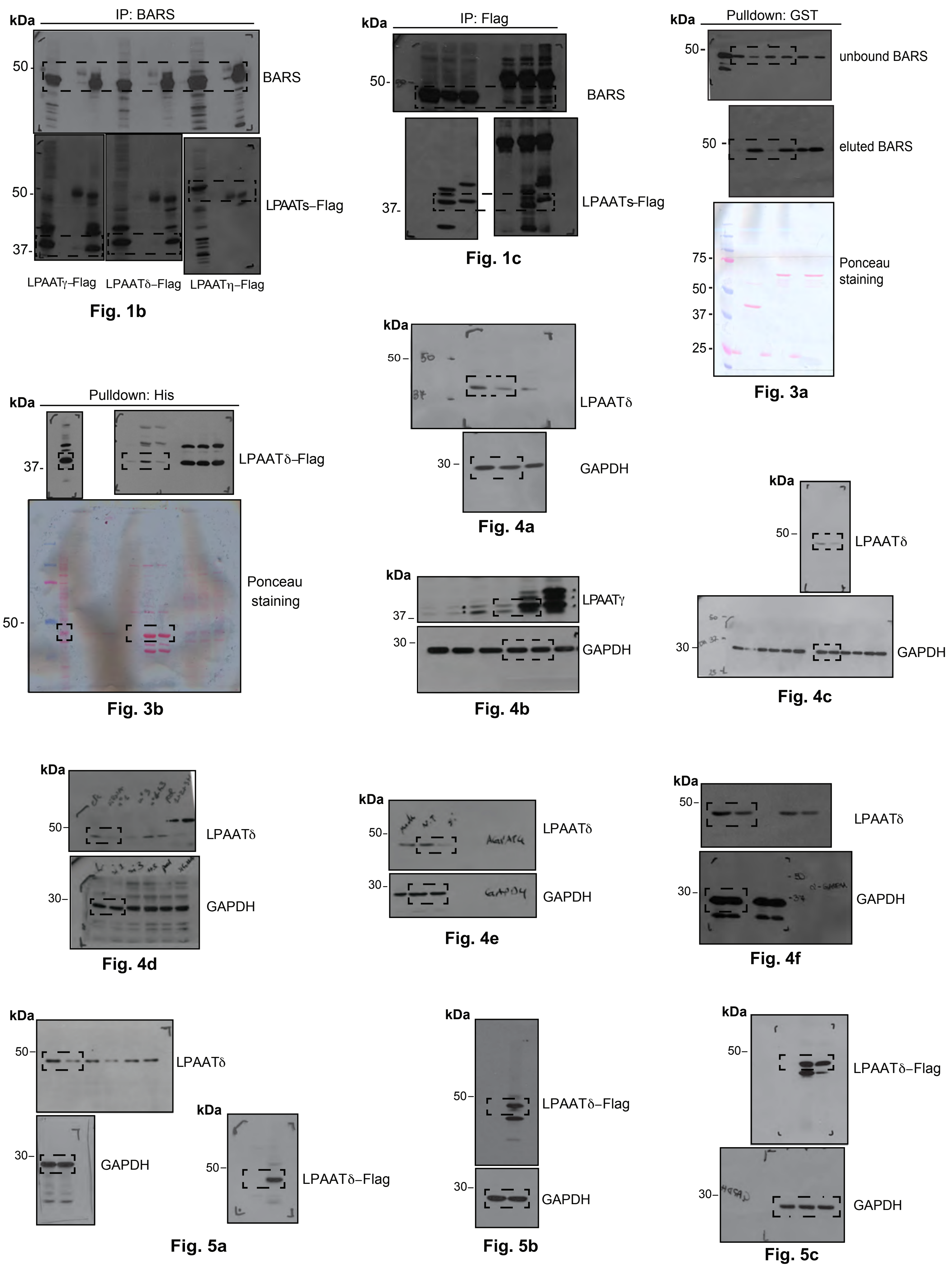

Supplementary Figure 11. Full scan images of all gel/Western blotting data.

Supplementary Fig.12

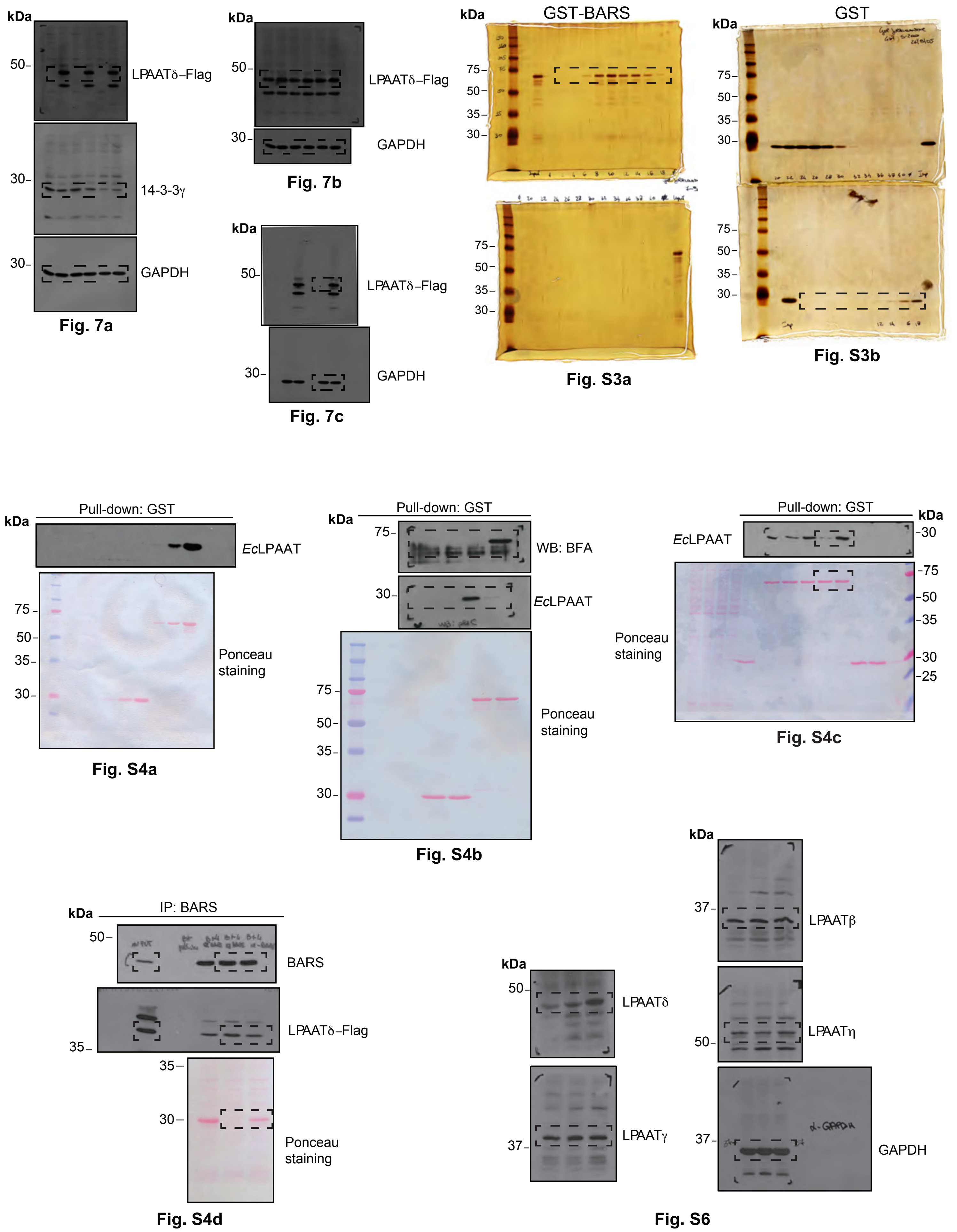

Supplementary Figure 12. Full scan images of all gel/Western blotting data.

Supplementary Fig.13

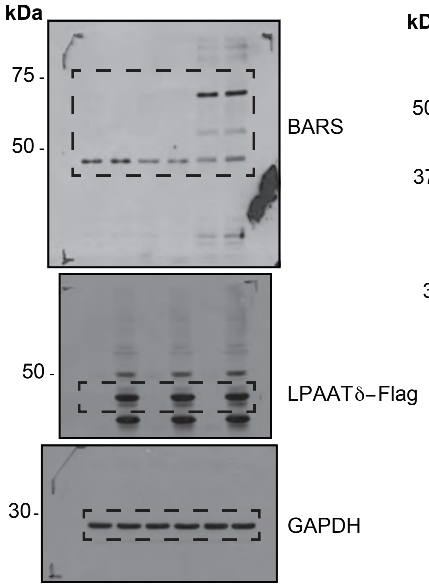

Fig. S7a

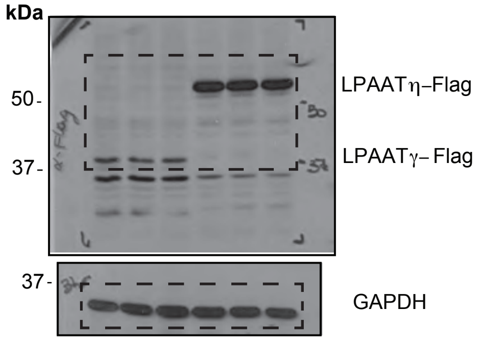

Fig. S7b

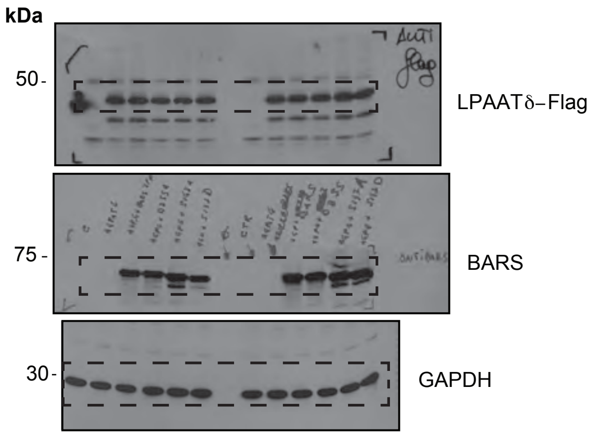

Fig. S7c

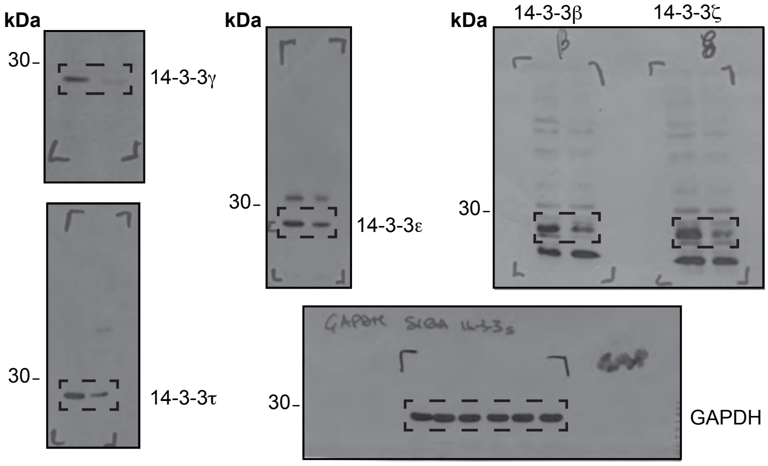

Fig. S9

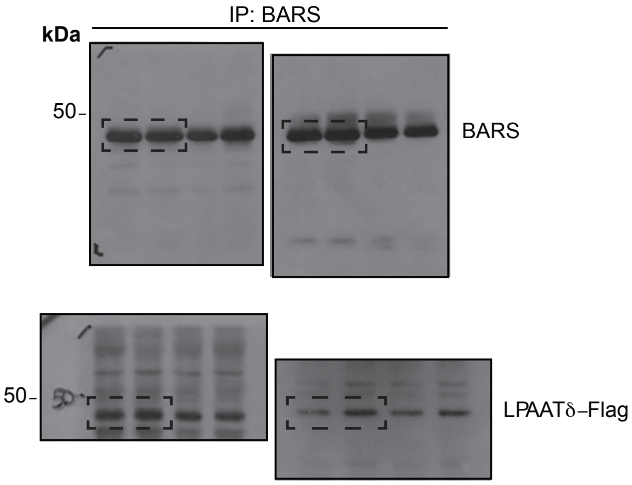

Fig. S8

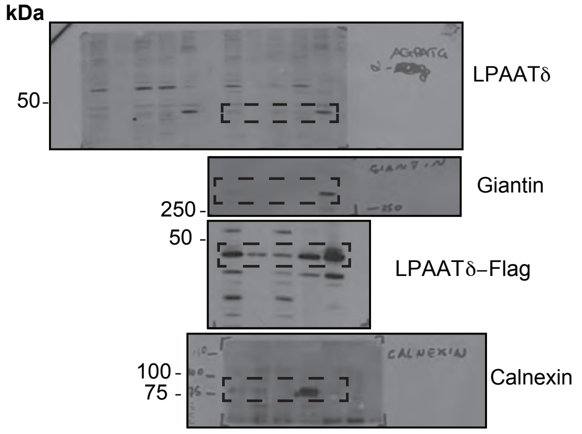

Fig. S10a

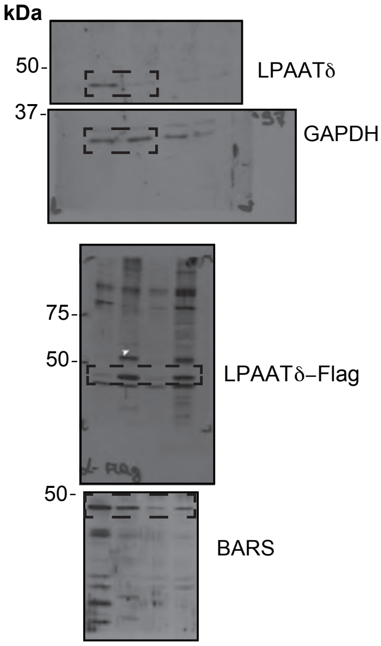

Fig. S10d

Supplementary Figure 13. Full scan images of all gel/Western blotting data.

TEMPLATE	OLIGONUCLEOTIDES	DIGESTIONS OF AMPLIFIED INSERTS	DESTINATION VECTOR
Human LPAATβ cDNA from Imagenes GmbH as the p0TB7 vector	5’-cggaattcatggagctgtggccgtgtct-3’ and 5’-gtggatcccctgggccggctgcacgcc-3’	BamH1 and EcoR1	BamH1/EcoR1-digested p3xFLAG CMV plasmid (SIGMA)
Human LPAATγ cDNA from Imagenes GmbH as the pBluescriptR	5’-gtagaattcaccatgggactgct-3’ and 5’-cgcgatatcccttcctttttcttaaactcttggt-3’	EcoR1 and ECOR5	Ecor1/EcoR5-digested p3xFLAG-CMV plasmid **(SIGMA)**
Human LPAATδ cDNA from Imagenes GmbH as the p0TB7 vector	5’-cggaattcatggacctggcgggactg-3’ and 5’-cgagatctggtcattcagtttctgcttg-3’	EcoR1 and Bgl2	Ecor1/BamH1-digested p3xFLAG-CMV plasmid **(SIGMA)**
Human LPAATε cDNA from Imagenes GmbH as the pSPORT1 vector	5’-cggaattcatgctgctgtccctggtg-3’ and 5’-gtggatccctgctttaatagtaacccacag-3’	EcoR1 and BamH1	EcoR1/BamH1-digested p3xFLAG-CMV plasmid **(SIGMA)**
Human LPAATζ cDNA from Imagenes GmbH as the p0TB7 vector	5’-cggaattcatgttcctgttgctgcctttt-3’ and 5’-cgagatctgggagcggctcctgtcctt-3’	EcoR1 and Bgl2	EcoR1/BamH1-digested p3xFLAG-CMV plasmid **(SIGMA)**
Human LPAATη cDNA from Imagenes GmbH as the pCMV-SPORT6 vector	5’-cggaattcatgagccagggaagtccgg-3’ and 5’-cgagatctggtctcccttctgcttgggt-3’	EcoR1 and Bgl2	EcoR1/Bgl2-digested p3xFLAG-CMV plasmid **(SIGMA)**
C-terminally Flag tagged LPAAT**δ** encoding the silent mutation	5’-cctgattcactgtgagggcacgaggttcactgaaaagaagcat gagatcagca-3’ and 5’-tgctgatctcatgcttcttttcagtgaacctcgtgccctcacagtgaatcagg-3’		
C-terminally Flag tagged LPAAT**δ**^{H96V}	5’-gccatcgtggttctcaacgtcaagtttgaaattgactttctgtg-3’ and 5’-cacagaaagtcaatttcaaacttgacgttgagaaccacgatggc-3’		
C-terminally CFP tagged LPAAT**δ**^{H96V} encoding the silent mutation	5’-cctgattcactgtgagggcacgaggttcactgaaaagaagcat gagatcagca-3’ and 5’-tgctgatctcatgcttcttttcagtgaacctcgtgccctcacagtgaatcagg-3’		
C-terminally CFP tagged LPAAT**δ**	5’-cggaattcatggacctggcgggactg-3’ 5’-ttaggtacctggtcattcagtttctgcttg-3’	EcoR1-Kpn1	EcoR1/Kpn1-digested pECFPN1
N-terminally YFP tagged BARS wt and Mutants encoding the silent mutation	5’-tcaatgacttcacagtcaaacaaatgaggcaaggagccttcctggtga-3’ and 5’-tcaccaggaaggctccttgcctcatttgtttgactgtgaagtcattga-3’		

Supplementary Table 1: List of the oligonucleotide sequences for amplification of the templates, and of the restriction enzymes used in the cloning of the listed expression vectors.
